# Unveiling the Diversity of Immunoglobulin Heavy Constant Gamma (*IGHG*) Gene Segments in Brazilian Populations Reveals 28 Novel Alleles and Evidence of Gene Conversion and Natural Selection

**DOI:** 10.3389/fimmu.2019.01161

**Published:** 2019-06-04

**Authors:** Verónica Calonga-Solís, Danielle Malheiros, Marcia Holsbach Beltrame, Luciana de Brito Vargas, Renata Montoro Dourado, Hellen Caroline Issler, Roseli Wassem, Maria Luiza Petzl-Erler, Danillo G. Augusto

**Affiliations:** ^1^Laboratório de Genética Molecular Humana, Departamento de Genética, Universidade Federal do Paraná, Curitiba, Brazil; ^2^Laboratório de Interação Planta-Bactéria, Departamento de Genética, Universidade Federal do Paraná, Curitiba, Brazil

**Keywords:** IGHG genes, immunoglobulin heavy chain, molecular characterization, genetic diversity, populations, DNA sequencing

## Abstract

Even though immunoglobulins are critical for immune responses and human survival, the diversity of the immunoglobulin heavy chain gene (*IGH*) is poorly known and mostly characterized only by serological methods. Moreover, this genomic region is not well-covered in genomic databases and genome-wide association studies due to particularities that impose technical difficulties for its analysis. Therefore, the *IGH* gene has never been systematically sequenced across populations. Here, we deliver an unprecedented and comprehensive characterization of the diversity of the *IGHG1, IGHG2*, and *IGHG3* gene segments, which encode the constant region of the most abundant circulating immunoglobulins: IgG1, IgG2, and IgG3, respectively. We used Sanger sequencing to analyze 357 individuals from seven different Brazilian populations, including five Amerindian, one Japanese-descendant and one Euro-descendant population samples. We discovered 28 novel *IGHG* alleles and provided evidence that some of them may have been originated by gene conversion between common alleles of different gene segments. The rate of synonymous substitutions was significantly higher than the rate of the non-synonymous substitutions for *IGHG1* and *IGHG2* (*p* = 0.01 and 0.03, respectively), consistent with purifying selection. Fay and Wu's test showed significant negative values for most populations (*p* < 0.001), which indicates that positive selection in an adjacent position may be shaping *IGHG* variation by hitchhiking of variants in the vicinity, possibly the regions that encode the Ig variable regions. This study shows that the variation in the *IGH* gene is largely underestimated. Therefore, exploring its nucleotide diversity in populations may provide valuable information for comprehension of its evolution, its impact on diseases and vaccine research.

## Introduction

Immunoglobulins (Ig) are glycoproteins produced exclusively by activated B-lymphocytes and plasma cells that mediate humoral response against pathogens. Each B-cell clone presents an antigen-specific membrane-bound immunoglobulin that, together with CD79A and CD79B molecules, comprise the B-cell receptor (BCR). After stimulation by antigens, B-cells secrete immunoglobulins (antibodies) with the same antigen-binding sites than the membrane-bound molecules.

All Ig share a similar basic structure composed of four polypeptide chains: two identical heavy chains and two identical light chains. The heavy chain has one variable domain (V_H_) and three or four constant domains (C_H_1, C_H_2, C_H_3, and C_H_4). Each light chain exhibits one variable domain (V_L_) and one constant domain (C_L_). The variable region (V_H_ and V_L_) is responsible for antigen recognition and binding while the constant regions (C_H_ and C_L_) primarily mediate the Ig effector functions, which includes complement activation and Fc Receptor binding (1, 2).

In humans, the immunoglobulin heavy chain gene (*IGH*) is located in chromosome region 14q32 and consists of four groups of gene segments: the variable heavy (*IGHV*), diversity heavy (*IGHD*), joining heavy (*IGHJ*), and constant heavy (*IGHC*). The *IGHC* group includes *IGHM, IGHD, IGHG3, IGHG1, IGHEP1, IGHA1, IGHGP, IGHG2, IGHG4, IGHGE*, and *IGHA2* gene segments and pseudogenes. Immunoglobulin light chains are encoded by two different genes: lambda (*IGL*) at 22q11 and kappa (*IGK*) at 2p11.2 (3, 4).

During B-cell development, the *IGH* gene undergoes a somatic rearrangement, in which only one *IGHV*, one *IGHD*, and one *IGHJ* gene segment are combined to form the Ig variable region V_H_. In contrast, during clonal expansion after activation of the B-cell, *IGHC* gene segments go through a process called class-switch recombination, which determines the Ig class and subclass: IgM, IgD, IgG (IgG1, IgG2, IgG3, and IgG4), IgA (IgA1 and IgA2), and IgE. The human humoral immune response is mainly mediated by Ig gamma (IgG), which is subdivided into four subclasses, IgG1, IgG2, IgG3, and IgG4, ordered by decreasing abundance in peripheral blood (5). The constant regions of these four subclasses are encoded by the gene segments *IGHG1, IGHG2, IGHG3*, and *IGHG4*, respectively, the first three being the ones focused on this study. Each *IGHG* gene segment consists of three exons that encode the constant heavy domains (*CH1, CH2*, and *CH3*) and exon *H*, which encodes the hinge between the CH1 and CH2 domains (5).

Most of the human IgG diversity in populations has only been characterized by serological methods, which defined the immunoglobulin allotypes at the protein level. Ig allotypes are polymorphic epitopes (resulting from nucleotide variation) on the Ig constant domain that provide binding sites for antibodies (6). Certain IgG allotypes have been associated with susceptibility to cancer, autoimmune and infectious diseases (7–9).

Although the genetic variability of some *IGHG* gene segments has been characterized (10, 11), it has never been systematically sequenced at the nucleotide level across populations. Thus, the diversity of these gene segments is probably underestimated. Additionally, this genomic region is not well-covered in genome-wide studies and genomic databases for two reasons: first, DNA samples used are often extracted from B-cell lines, which are not suitable for analyzing this region due to the somatic rearrangement within this locus; second, the high sequence similarity of these segments imposes technical difficulties for sequencing and genotyping (12).

Here, we analyzed the diversity of *IGHG1, IGHG2*, and *IGHG3* in seven Brazilian populations: five Amerindian populations that have been genetically isolated for centuries and two urban populations. By analyzing deep sequencing data, we found 28 novel *IGHG* alleles, characterized the linkage disequilibrium of variants within these segments and analyzed the relationship among alleles. Additionally, we provided compelling evidence of the occurrence of gene conversion between different gene segments and evidence of purifying selection shaping *IGHG* diversity.

## Methods

### Characterization of the Study Populations

**Figure 1:**
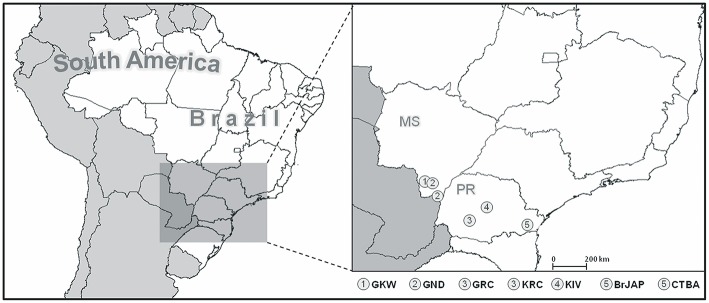
Location of the study populations. KIV, Kaingang from Ivaí; KRC, Kaingang from Rio das Cobras; GRC, Guarani Mbya; GKW, Guarani Kaiowa; GND, Guarani Ñandeva; BrJAP, Japanese-descendant from Curitiba; CTBA, Euro-descendants from Curitiba.

This study was approved by the Brazilian National Human Research Ethics Committee (CONEP), protocol number CAAE 02727412.4.0000.0096, in accordance to the Brazilian Federal laws. We analyzed a total of 357 individuals from seven Brazilian populations, of which five are Amerindian: Guarani Kaiowa (GKW, *n* = 46), Guarani Ñandeva (GND, *n* = 48), Guarani Mbya (GRC, *n* = 51), Kaingang from Ivaí (KIV, *n* = 52), and Kaingang from Rio das Cobras (KRC, *n* = 52); and two are urban populations: Japanese-descendants (BrJAP, *n* = 57) and Euro-descendants from Curitiba (CTBA, *n* = 51). Their detailed geographic location and sample sizes are found in Figure 1 and Table S1.

The Amerindians samples were collected between late 1980s and early 1990s. According to public data from the Brazilian Institute of Geography and Statistics (IBGE), there are approximately 900,000 Amerindians individuals in Brazil, distributed across 693 official indigenous lands (https://www.ibge.gov.br). The Guarani speak a Tupi-Guarani language, which belongs to the Tupi language family. The Kaingang speak Jê, which belongs to the Macro-Jê language stock. Analyzing mtDNA segments and the proposed time of origin of Tupi-Guarani and Jê linguistic families, Marrero and colleagues (13) estimated the Guarani population split in three partialities (Guarani Kaiowa, Guarani Ñandeva and Guarani Mbya) 1,800 years ago, while the different Kaingang populations would have split more recently, around 200 years ago. Since then, they are believed to have remained isolated from each other and other urban populations due to strong cultural and language barriers (14). A former study from our group estimated that the gene flow of these Amerindian populations with non-Amerindians was low, being 0% in Guarani Kaiowa, 4% in Guarani Mbya, 14% in Guarani Ñandeva and 7% in Kaingang (15).

The two urban samples were from Curitiba, the capital of Paraná State and the 5th largest city in Brazil. As a result of the Brazilian history of European colonization 500 years ago, and especially the more recent European migrations since the 19^th^ Century, the population of Curitiba is of predominantly European ancestry. According to the public data from IBGE, 78.7% of the inhabitants of Curitiba self-declared themselves as white, 16.7% as admixed, 3% as black, 1.4% as Asian, and 0.2% as Amerindian (https://www.ibge.gov.br). The population here referred as CTBA only included Euro-descendant individuals. Therefore, we excluded all individuals with known miscegenation with Amerindian and/or other non-European ancestries. Paraná State also hosts the second largest Japanese community in Brazil, one of the largest outside Japan. The Japanese migration started in the twentieth century with the Treaty of Friendship, Commerce and Navigation between Brazil and Japan. All Japanese-descendent individuals of this study (BrJAP) were born in Brazil, with either both parents or all four grandparents born in Japan. They reported no history of admixture with non-Japanese ethnicities.

### DNA Extraction

Genomic DNA was extracted from peripheral blood samples by standard salting-out (16) or by the phenol-chloroform-isoamyl method (17). High-quality DNA has been stored at −80°C since the extraction. DNA integrity was evaluated by 1% agarose gel electrophoresis and purity was accessed by spectrophotometry.

### Sequencing and Allele Identification

We aligned all previously known *IGHG* alleles and designed primers to amplify each segment specifically. To define the best set of primers we used the following approaches: (i) we ruled out unspecific amplification by verifying that all amplicons did not exhibit any variant that was specific of other segments; (ii) we certified that the genotype distribution of all single nucleotide variable sites, in each amplicon, were in accordance to Hardy-Weinberg equilibrium (*p* > 0.05).

Polymerase chain reaction (PCR) was performed for *IGHG1, IGHG2*, and *IGHG3* as follows: 1X Buffer (Invitrogen); 0.2 mM dNTP (Life Technologies); 1.5 mM MgCl_2_ (Invitrogen, Carlsbad, CA, USA); 0.3 μM of each primer; 0.05 U/μL Taq polymerase Platinum® (Invitrogen, Carlsbad, CA, USA); and 2 ng/μL genomic DNA. The segments were amplified in a Mastercycler ep Gradient S thermocycler (Eppendorf, Hamburg, Germany), with a first step at 94°C for 2 min and 10 cycles of 94°C for 15 s, T_m_A °C for 15 s and 72°C for 60 s, followed by 25 cycles of 94°C for 15 s, T_m_B °C for 15 s and 72°C for 60 s, with a final extension step of 72°C for 60 s (primer sequences, location, and amplification temperatures are available in Table S2 and Figure S1). Amplicons were visualized by 1% agarose gel electrophoresis with 1% UniSafe Dye® (Uniscience, Sao Paulo, Brazil). Afterwards, PCR products were purified with 0.8 U/μL of exonuclease I enzyme (Fermentas, Waltham, MA, EUA) and 0.14 U/μL of alkaline phosphatase (ThermoFisher Scientific, Waltham, MA, EUA).

Sequencing was performed using Big Dye® Terminator Cycle Sequencing Standard v3.1 (Life Technologies, Carlsbad, CA, USA), according to manufacturer's instructions. The sequencing reactions were performed in a Mastercycler ep Gradient S thermocycler (Eppendorf, Hamburg, Germany) with a first step at 95°C for 60 s and 25 cycles of 95°C for 10 s, 50°C for 5 s, and 60°C for 4 min, followed by capillary electrophoresis in a 3500xl Genetic Analyzer Sequencer (Life Technologies, Carlsbad, CA, USA).

After sequencing, the alleles were identified according to the known alleles described at IMGT database (International ImMunoGeneTics Information System) (18). IMGT database provides public access to an integrated information system specialized in immunoglobulins (Ig), T cell receptors (TCR), and major histocompatibility complex (MHC) genes and molecules. All data submitted to the IMGT database are manually checked by experts in the field, which assure the deposit of high-quality data.

The nucleotide sequence of each individual was aligned with consensus sequences with Mutation Surveyor® DNA Variant Analysis Software v5.0.1 (Softgenetics), and their variable sites were annotated. Alleles that were different from the ones listed in the IMGT database were considered novel and were subsequently confirmed by sequencing and/or molecular cloning as described below.

The novel alleles that were observed in homozygosis (IGHG1^*^07, IGHG1^*^08, IGHG2^*^09, IGHG2^*^13, IGHG3^*^21, IGHG3^*^22, IGHG3^*^26) were confirmed by direct re-sequencing from a different PCR product. Novel alleles observed in heterozygosis without phasing ambiguities due to the presence of only one heterozygous position (IGHG1^*^06, IGHG1^*^09, IGHG1^*^10, IGHG1^*^12, IGHG1^*^13, IGHG1^*^14, IGHG2^*^07, IGHG2^*^10, IGHG2^*^12, IGHG3^*^20, IGHG3^*^27, IGHG3^*^28) were also confirmed by re-sequencing. The new variants with ambiguous phasing (IGHG1^*^11, IGHG2^*^08, IGHG2^*^11, IGHG2^*^14, IGHG2^*^15, IGHG3^*^23, IGHG3^*^24, IGHG3^*^25, IGHG3^*^29) were confirmed by molecular cloning. In this case, the segments were re-amplified and ligated into a PTZ57R/T vector (Fermentas, Waltham, MA, EUA) with terminal deoxynucleotidyl transferase (TdT) enzyme. Afterwards, recombinant plasmids were obtained and purified from multiple transformed colonies and sequenced as described above. Novel alleles were verified based on sequences from at least two independent colonies containing each allele.

### Data Analysis

Allelic frequencies were obtained by direct counting using GenAlEx v6.502 software (19). Hardy-Weinberg equilibrium was tested for each gene segment in all populations by Guo and Thompson's method (20), performed in Arlequin v3.5.2 software (21). *IGHG* haplotypes from different gene segments were estimated via ELB algorithm and this information was used for Gm allotype haplotype inference, according to the correspondence between nucleotide variants and allotypes described by Lefranc et al. (6). Linkage disequilibrium (LD) between single nucleotide variants of each gene segment was estimated with Haploview software (22).

Allele networks were performed with variants from each gene segment through the median-joining (MJ) algorithm (23) with Network v5.0 software. Allele frequencies were compared using the exact test of population differentiation (24) and population-pairwise F_ST_ (25, 26) with Arlequin v3.5.2 software (21). Principal component analysis (PCA) was performed using the Minitab 17 Statistics Software (27) for graphical representation of the genetic differences and similarities in the major components of variation among populations. The PCA was performed using inferred allotype haplotype frequencies to compare the frequencies from the study population with others that were previously described serologically. These haplotypes were classified according to Lefranc et al. (6), and detailed information is available in Table S3.

Neutrality tests were performed using the Tajima's *D* (28), Fu and Li's D^*^, F^*^, D, and *F* (29) and Fay and Wu's *H* (30) in DnaSP software (31). Homologous gene segments from rhesus monkey were used as outgroup *(Macaca mulatta;* accession number: NW_001121238, AY292519, AY292512).

## Results

### One Novel Single Nucleotide Variant and 28 Novel *IGHG* Alleles Have Been Discovered

Within all three gene segments in the seven populations analyzed, we found a total of 49 exonic variable sites, of which 26 were non-synonymous substitutions. Based on the Grantham scale (32), which ranges from 5 to 215 according to the physicochemical distance between amino acid pairs, amino acid replacements were from low to moderate (15 < D < 103) (Table 1). Of the single variable sites, 21 have not been reported in any of the previously described alleles at the IMGT database (Table 1, in bold). We also found a novel synonymous *IGHG3* single nucleotide variant at the position chr14:106235856 (GRCh37.p13 primary assembly) in the CTBA population. This new variant was submitted to the dbSNP database (34) under reference SNP ID number *rs155533833* (NC_000014.8:g.106235856G>A).

**Table 1:** Variable sites found in IGHG1, IGHG2, and IGHG3 gene segments.

**Gene segment**	**Exon**	**rsID**	**Location**^**a**^	**IMGT numbering**^**b**^	**Eu numbering**^**c**^	**Nucleic acid substitution**	**Amino acid substitution**	**Allotype**	**Grantham's *D***^**d**^	**Frequency**^**e**^
*IGHG1*	CH1	rs11552998	106209340	19	140	G>A				0.006
		**rs17850096**	106209289	40	157	G>C				0.001
		rs1071803	106209119	120	214	A>G	K>R	Gm17>Gm3	26	0.143
	CH2	**rs587690960**	106208471	22	260	A>G				0.003
		**rs377538050**	106208364	84.3	296	A>T	Y>F		22	0.001
		**rs193160354**	106208327	91	308	C>T				0.011
		**rs1043109**	106208326	92	309	C>G	L>V		32	0.011
		**rs1043249**	106208306	98	315	T>C				0.011
	CH3	**rs11557940**	106208107	5	349	C>T				0.001
		rs1045853	106208086	12	356	T>G	D>E	Gm1>nGm1	45	0.118
		rs11621259	106208082	14	358	C>A	L>M	Gm1>nGm1	15	0.118
		rs17841087	106207933	86	407	C>T				0.114
		rs113804727	106207862	110	431	C>G	A>G	nGm2>Gm2	60	0.270
		**rs370028332**	106207858	112	432	G>C				0.001
		rs8011686	106207843	117	437	G>A				0.003
		rs12879979	106207822	124	444	T>C				0.106
*IGHG2*	CH1	**rs189328740**	106111071	15	136	C>T				0.023
		**rs587648672**	106111069	16	137	A>G	E>G		98	0.023
		**rs773818177**	106111067	17	138	A>G	S>G		56	0.023
		rs11557955	106110966	82	171	A>G				0.157
		rs11627594	106110914	92	189	C>A	P>T		38	0.105
	CH2	rs8009156	106110137	45.1	282	G>A	V>M	Gm(.)>Gm23	21	0.103
		rs11160859	106110057	91	308	T>C				0.163
		**rs113678609**	106110056	92	309	G>C	V>L		32	0.003
	CH3	**rs587682450**	106109825	9	353	A>C				0.017
		rs4983499	106109752	38	378	G>T	A>S		99	0.003
		**rs368359789**	106109708	79	392	G>C	K>N		94	0.001
		rs1049810	106109702	81	394	A>G				0.054
		rs28371022	106109573	117	437	G>A				0.106
*IGHG3*	CH1	rs2983777	106237642	30	151	C>A				0.001
		**rs12050095**	106237624	40	157	G>A				0.025
	CH2	**rs138869693**	106236202	35	271	C>T				0.006
		**rs145035200**	106236195	38	274	C>A	Q>K		53	0.006
		rs74093865	106236143	82	291	C>T	P>L	nGm21>Gm21	98	0.797
		rs60746425	106236141	83	292	C>T	R>W	nGm16>Gm16	101	0.048
		rs12890621	106236128	84.3	296	A>T	Y>F		22	0.123
		**rs201027762**	106236035	110	327	C>G	A>G		60	0.006
		rs141959627	106236000	124	339	A>G	T>A		58	0.006
	CH3	rs189025987	106235895	1.4	341	A>G				0.001
		**rs147594653**	106235874	4	348	G>A				0.001
		*rs155533833	106235856	10	354	C>T				0.001
		rs113169458	106235783	39	379	G>A	V>M	nGm15>Gm15	21	0.047
		rs77307099	106235767	44	384	G>A	S>N	Gm11>nGm11	46	0.799
		rs78376194	106235766	44	384	C>T		Gm11>nGm11		0.799
		**rs587739524**	106235758	45.2	387	C>G	P>R		103	0.003
		rs149653267	106235742	79	392	C>G	N>K		94	0.052
		rs139413052	106235729	84	397	A>G	M>V	Gm14>nGm14	21	0.048
		rs4042056	106235614	115	435	G>A	R>H	Gm5>nGm5	29	0.085
		rs1051112	106235611	116	436	T>A	F>Y	Gm5>nGm5	22	0.847

In bold, variant sites that have not been observed in any allele listed in the IMGT database. ^*^Novel single nucleotide variation submitted to dbSNP database.

a Coordinate at chromosome 14 location (GRCh37.p13 primary assembly).

b Amino acid position according to IMGT database (International ImMunoGeneTics Information System) (18).

c According Edelman et al. (33).

d Physicochemical distances between amino the amino acids involved in the substitution, according Grantham (32). The higher the value, the greater the differences, ranging from 5 to 215.

e *Frequency of the alternative allele, merging all the samples of this study*.

A total of 28 novel *IGHG* alleles have been found in our study: nine in *IGHG1* (Table 2), nine in *IGHG2* (Table 3), and ten in *IGHG3* (Table 4). All novel alleles have been confirmed either by sequencing or by molecular cloning followed by sequencing. Novel alleles have been submitted to IMGT Nomenclature Committee (18), which verified the accuracy of our data and assigned official names (reports #2018-2-0824 and #2018-5-1113).

**Table 2:** *IGHG1* alleles previous described and the 9 novel *IGHG1* alleles identified in this study.

		**Exon**	**CH1**			**CH2**						**CH3**									
		**IMGT unique numbering**	19	40	120	22	84.3	85,1	91	92	98	5	12	14	86	101	110	112	117	124	
		**Eu numbering**^**a**^	140	157	214	260	296	301	308	309	315	349	356	358	407	422	431	432	437	444	
		**Amino acid change**	A	S	K>R	T	Y>F	R	V	L>V	N	Y	D>E	L>M	Y	C>I	A>G	L	T	S	
		**Exonic position**	68	119	289	89	196	212	233	234	254	26	47	51	200	243	271	275	290	311	
		**Consensus nucleotide**	G	G	A	A	A	C	C	C	T	C	T	C	C	G	C	G	G	T	
**Allele name**	**GenBank accession number**	**Allotype**^**b**^																			**#**
IGHG1*01	–	17,1	.	.	.	.	.	.	.	.	.	.	.	.	.	.	.	.	.	.	0
IGHG1*02	–	17,1	.	.	.	.	.	T	.	.	.	.	.	.	.	.	.	.	.	.	391
IGHG1*03	–	3	.	.	G	.	.	T	.	.	.	.	G	A	T	.	.	.	.	C	81
IGHG1*04	–	17,1,27	.	.	.	.	.	T	.	.	.	.	.	.	.	A	.	.	.	.	0
IGHG1*05	–	17,1	A	.	.	.	.	T	.	.	.	.	.	.	.	.	.	.	A	.	2
IGHG1*06	**MG920252**	**3**	.	.	G	.	.	T	.	.	.	.	G	A	T	.	.	C	.	C	1
IGHG1*07	**MG920245**	**17,1,2**	.	.	.	.	.	T	.	.	.	.	.	.	.	.	G	.	.	.	189
IGHG1*08	**MG920246**	**3,1**	.	.	G	.	.	T	.	.	.	.	.	.	.	.	.	.	.	.	18
IGHG1*09	**MG920247**	**17,1**	A	.	.	.	.	T	.	.	.	.	.	.	.	.	.	.	.	.	2
IGHG1*10	**MG920248**	**17,1**	.	.	.	G	.	T	.	.	.	.	.	.	.	.	.	.	.	.	2
IGHG1*11	**MG920249**	**17,1**	.	.	.	.	.	T	T	G	C	.	.	.	.	.	.	.	.	.	7
IGHG1*12	**MG920250**	**17,1**	.	.	.	.	.	T	.	.	.	T	.	.	.	.	.	.	.	.	1
IGHG1*13	**MG920251**	**17,1**	.	.	.	.	T	T	.	.	.	.	.	.	.	.	.	.	.	.	1
IGHG1*14	**MG920253**	**17,1**	.	C	.	.	.	T	.	.	.	.	.	.	.	.	.	.	.	.	1

Novel alleles (in bold) have been confirmed by sequencing and/or molecular cloning. Their official names have been assigned by IMGT nomenclature committee. IMGT, International ImMunoGeneTics Information System (18). Dots represent the consensus nucleotide.

a According to Edelman et al. (33).

b llotypes were inferred according to Lefranc et al. (6).

# *Number of copies observed in this study*.

**Table 3:** *IGHG2* alleles previous described and the 9 novel *IGHG2* alleles identified in this study.

		**Exon**	**CH1**								**CH2**			**CH3**					
		**IMGT unique numbering**	15	16	17	19	82	92	95	96	45.1	91	92	9	38	79	81	117	
		**Eu numbering**^**a**^	136	137	138	140	171	189	192	193	282	308	309	353	378	392	394	437	
		**Amino acid change**	S	E>G	S>G	A	P	P>T	N>S	F>L	V>M	V	V>L	P	A>S	K>N	T	T	
		**Exonic position**	56	58	60	68	161	213	223	227	150	230	231	38	111	155	161	290	
		**Consensus nucleotide**	C	A	A	C	A	C	A	C	G	T	G	A	G	G	A	G	
**Allele name**	**GenBank accession number**	**Allotype**^**b**^																	**#**
IGHG2*01		(..)	.	.	.	.	.	.	.	.	.	.	.	.	.	.	.	.	0
IGHG2*02		23	.	.	.	G	G	A	.	.	A	C	.	.	.	.	.	A	71
IGHG2*03		(..)	.	.	.	G	.	.	.	.	.	.	.	.	.	.	.	.	557
IGHG2*04		(..)	.	.	.	G	.	.	G	G	.	.	.	.	.	.	.	.	0
IGHG2*05		(..)	.	.	.	G	.	.	.	.	.	.	.	.	.	.	G	.	0
IGHG2*06		(..)	.	.	.	G	G	.	.	.	.	C	.	.	T	.	.	A	2
IGHG2*07	**MH025828**	**(..)**	.	.	.	G	.	.	.	.	.	C	.	.	.	.	.	.	4
IGHG2*08	**MH025829**	**(..)**	.	.	.	G	G	.	.	.	.	C	.	.	.	.	G	.	30
IGHG2*09	**MH025830**	**(..)**	T	G	G	G	.	.	.	.	.	.	.	.	.	.	.	.	16
IGHG2*10	**MH025831**	**(..)**	.	.	.	G	.	.	.	.	.	.	.	C	.	.	.	.	12
IGHG2*11	**MH025832**	**(..)**	.	.	.	G	G	.	.	.	.	C	C	.	.	.	G	.	1
IGHG2*12	**MH025833**	**(..)**	.	.	.	G	G	.	.	.	.	.	.	.	.	.	.	.	3
IGHG2*13	**MH025834**	**(..)**	.	.	.	G	.	.	.	.	.	C	.	.	.	.	G	.	8
IGHG2*14	**MH025835**	**(..)**	.	.	.	G	G	.	.	.	.	C	C	.	.	.	.	.	1
IGHG2*15	**MH025836**	**(..)**	.	.	.	G	G	.	.	.	.	C	.	.	.	C	G	.	1

Novel alleles (in bold) have been confirmed by sequencing and/or molecular cloning. Their official names have been assigned by IMGT nomenclature committee. IMGT, International ImMunoGeneTics Information System (18). Dots represent the consensus nucleotide.

a *According to Edelman et al. (33)*.

b *Allotypes were inferred according to Lefranc et al. (6)*.

# *Number of copies observed in this study*.

**Table 4:** *IGHG3* alleles previous described and the 10 novel *IGHG3* alleles identified in this study.

		**Exon**	**CH1**						**H1**	**H2**	**H3**	**H4**		**CH2**								
		**Eu numbering**^**a**^	**118**	**151**	**157**	**176**	**192**	**193**			**-**	**-**	**-**	**271**	**274**	**291**	**292**	**296**	**309**	**327**	**339**	
		**IMGT unique numbering**	**1.4**	**30**	**40**	**84,3**	**95**	**96**			**10**	**10**	**13**	**35**	**38**	**82**	**83**	**84,3**	**92**	**110**	**124**	
		**Amino acid change**	**S**	**S**	**S**	**S>Y**	**S>N**	**L>F**			**P**	**P**	**R**	**P**	**Q>K**	**P>L**	**R>W**	**Y>F**	**L>V**	**A>G**	**T>A**	
		**Exonic position**	**2**	**101**	**119**	**175**	**223**	**227**			**29**	**29**	**36**	**122**	**129**	**181**	**183**	**196**	**234**	**289**	**324**	
		**Consensus nucleotide**	**T**	**C**	**G**	**C**	**G**	**G**			**A**	**G**	**A**	**C**	**C**	**C**	**C**	**A**	**C**	**C**	**A**	
**Allele name**	**GenBank accession number**	**Allotype**^**b**^																				**#**
IGHG3*01		5,10,11,13,14,26,27	.	.	.	.	.	.	.	.	.	.	.	.	.	.	.	.	.	.	.	2
IGHG3*03		5,6,11,24,26	.	.	.	.	.	.	.	abs	.	.	.	.	.	.	.	.	.	.	.	0
IGHG3*04		5,10,11,13,14,26,27	C	.	.	.	.	.	.	abs	abs	.	.	.	.	.	.	.	.	.	.	0
IGHG3*05		5,10,11,13,14,26,27	.	.	.	.	.	.	.	.	.	.	.	.	.	.	.	.	.	.	.	0
IGHG3*06		5,10,11,13,14,26,27	.	A	.	.	.	.	.	.	.	.	.	.	.	.	.	.	.	.	.	0
IGHG3*07		5,10,11,13,14,26,27	.	.	.	.	.	.	.	.	.	.	.	.	.	.	.	.	.	.	.	0
IGHG3*08		5,14,26,27	.	.	.	.	.	.	.	.	.	.	.	.	.	.	.	.	.	.	.	0
IGHG3*09		5,10,11,13,14,26,27	.	.	.	.	.	.	.	.	.	.	.	.	.	.	.	.	G	.	.	0
IGHG3*10		5,10,11,13,14,26,27	.	A	.	.	.	.	.	.	.	.	.	.	.	.	.	.	.	.	.	1
IGHG3*11		5,10,11,13,14,26,27	.	.	.	.	.	.	.	.	.	A	C	.	.	.	.	T	.	.	.	76
IGHG3*12		5,10,11,13,14,26,27	.	.	.	.	.	.	.	.	abs	A	C	.	.	.	.	T	.	.	.	7
IGHG3*13		5,6,10,11,14,26,27	.	.	.	.	.	.	.	.	.	.	.	.	.	.	.	.	.	.	.	0
IGHG3*14		21,26,27,28	.	.	.	.	.	.	.	.	.	.	.	.	.	T	.	.	.	.	.	529
IGHG3*15		21,26,27,28	.	.	.	.	.	.	.	.	.	.	.	.	.	T	.	.	.	.	.	1
IGHG3*16		21,26,27,28	.	.	.	.	.	.	.	.	.	.	.	.	.	T	.	.	.	.	G	5
IGHG3*17		10,11,13,15,27	.	.	.	.	A	C	.	abs	G	.	.	.	.	.	.	.	.	.	.	0
IGHG3*18		10,11,13,15,16,27	.	.	.	A	.	.	.	abs	G	.	.	.	.	.	T	.	.	.	.	0
IGHG3*19		10,11,13,15,16,27	.	.	.	.	.	.	.	abs	G	.	.	.	.	.	T	.	.	.	.	31
IGHG3*20	**MG920256**	**21,26,27,28**	.	.	.	.	.	.	.	.	.	.	.	.	.	T	.	.	.	.	.	2
IGHG3*21	**MG920255**	**5,10,11,13,14,26,27**	.	.	A	.	.	.	.	.	.	.	.	.	.	.	.	.	.	.	.	18
IGHG3*22	**MG920254**	**21,27**	.	.	.	.	.	.	.	.	.	.	.	.	.	T	.	.	.	.	.	26
IGHG3*23	**MH025837**	**10,11,13,16,27**	.	.	.	.	.	.	.	abs	G	.	.	.	.	.	T	.	.	.	.	1
IGHG3*24	**MG920257**	**26,27,28**	.	.	.	.	.	.	.	.	.	.	.	.	.	.	.	.	.	.	.	2
IGHG3*25	**MG920258**	**21,26,27,28**	.	.	.	.	.	.	.	.	.	.	.	T	A	T	.	.	.	.	.	4
IGHG3*26	**MG920259**	**5,10,11,13,14,26,27**	.	.	.	.	.	.	.	.	.	.	.	.	.	.	.	T	.	G	.	4
IGHG3*27	**MG920260**	**26,27,28**	.	.	.	.	.	.	.	.	.	.	.	.	.	T	.	.	.	.	.	1
IGHG3*28	**MG786813**	**5,10,11,13,14,26,27**	.	.	.	.	.	.	.	.	.	A	C	.	.	.	.	T	.	.	.	1
IGHG3*29	**MG920261**	**21,26,27,28**	.	.	.	.	.	.	.	.	.	.	.	.	.	T	.	.	.	.	.	1

**Table:** 

		**Exon**	**CH3**																		
		**IMGT unique numbering**	**1,4**	**4**	**10**	**39**	**44**	**44**	**45,2**	**79**	**81**	**84**	**88**	**89**	**90**	**98**	**100**	**101**	**115**	**116**	
		**Eu numbering**^**a**^	**341**	**348**	**354**	**379**	**384**	**384**	**387**	**392**	**394**	**397**	**409**	**410**	**411**	**419**	**421**	**422**	**435**	**436**	
		**Amino acid change**	**S**	**V**	**S**	**V>M**	**S>N**	**S>N**	**P>R**	**N>K**	**SIN**	**M>V**	**K>R**	**SIN**	**SIN**	**Q>E**	**SIN**	**I>V**	**R>H**	**F>Y**	
		**Exonic position**	**2**	**23**	**41**	**114**	**130**	**131**	**139**	**155**	**161**	**168**	**205**	**209**	**212**	**234**	**242**	**243**	**283**	**286**	
		**Consensus nucleotide**	**A**	**G**	**C**	**G**	**G**	**C**	**C**	**C**	**G**	**A**	**A**	**C**	**C**	**C**	**C**	**A**	**G**	**T**	
**Allele name**	**GenBank accession number**	**Allotype**^**b**^																			**#**
IGHG3*01		5,10,11,13,14,26,27	.	.	.	.	.	.	.	.	.	.	.	.	.	.	.	.	.	.	2
IGHG3*03		5,6,11,24,26	.	.	.	.	.	.	.	.	.	G	G	A	.	G	T	G	.	.	0
IGHG3*04		5,10,11,13,14,26,27	.	.	.	.	.	.	.	.	.	.	.	.	.	.	.	.	.	.	0
IGHG3*05		5,10,11,13,14,26,27	.	.	.	.	.	.	.	.	.	.	.	.	.	.	.	.	.	.	0
IGHG3*06		5,10,11,13,14,26,27	G	.	.	.	.	.	.	G	.	.	.	.	.	.	.	.	.	.	0
IGHG3*07		5,10,11,13,14,26,27	G	.	.	.	.	.	.	G	.	.	.	.	.	.	.	.	.	.	0
IGHG3*08		5,14,26,27	.	.	.	.	A	T	.	.	.	.	.	.	.	.	.	.	.	.	0
IGHG3*09		5,10,11,13,14,26,27	.	.	.	.	.	.	.	.	.	.	.	.	T	.	.	.	.	.	0
IGHG3*10		5,10,11,13,14,26,27	.	.	.	.	.	.	.	.	.	.	.	.	.	.	.	.	.	.	1
IGHG3*11		5,10,11,13,14,26,27	.	.	.	.	.	.	.	.	.	.	.	.	.	.	.	.	.	.	76
IGHG3*12		5,10,11,13,14,26,27	.	.	.	.	.	.	.	.	.	.	.	.	.	.	.	.	.	.	7
IGHG3*13		5,6,10,11,14,26,27	.	.	.	.	.	.	.	G	A	.	.	.	.	G	.	.	.	.	0
IGHG3*14		21,26,27,28	.	.	.	.	A	T	.	.	.	.	.	.	.	.	.	.	.	A	529
IGHG3*15		21,26,27,28	.	.	.	.	A	T	.	G	.	.	.	.	.	.	.	.	.	A	1
IGHG3*16		21,26,27,28	.	.	.	.	A	T	.	.	.	.	.	.	.	.	.	.	.	A	5
IGHG3*17		10,11,13,15,27	.	.	.	A	.	.	.	G	.	G	.	.	.	.	.	.	A	A	0
IGHG3*18		10,11,13,15,16,27	.	.	.	A	.	.	.	G	.	G	.	.	.	.	.	.	A	A	0
IGHG3*19		10,11,13,15,16,27	.	.	.	A	.	.	.	G	.	G	.	.	.	.	.	.	A	A	31
IGHG3*20	**MG920256**	**21,26,27,28**	.	.	.	.	A	T	G	.	.	.	.	.	.	.	.	.	.	A	2
IGHG3*21	**MG920255**	**5,10,11,13,14,26,27**	.	.	.	.	.	.	.	.	.	.	.	.	.	.	.	.	.	.	18
IGHG3*22	**MG920254**	**21,27**	.	.	.	.	A	T	.	.	.	.	.	.	.	.	.	.	A	A	26
IGHG3*23	**MH025837**	**10,11,13,16,27**	.	.	.	.	.	.	.	G	.	G	.	.	.	.	.	.	A	A	1
IGHG3*24	**MG920257**	**26,27,28**	.	.	.	.	A	T	.	G	.	.	.	.	.	.	.	.	.	A	2
IGHG3*25	**MG920258**	**21,26,27,28**	.	.	.	.	A	T	.	.	.	.	.	.	.	.	.	.	.	A	4
IGHG3*26	**MG920259**	**5,10,11,13,14,26,27**	.	.	.	.	.	.	.	.	.	.	.	.	.	.	.	.	.	.	4
IGHG3*27	**MG920260**	**26,27,28**	.	A	.	.	A	T	.	.	.	.	.	.	.	.	.	.	.	A	1
IGHG3*28	**MG786813**	**5,10,11,13,14,26,27**	.	.	T	.	.	.	.	.	.	.	.	.	.	.	.	.	.	.	1
IGHG3*29	**MG920261**	**21,26,27,28**	G	.	.	.	A	T	.	.	.	.	.	.	.	.	.	.	.	A	1

Novel alleles (in bold) have been confirmed by sequencing and/or molecular cloning. Their official names have been assigned by IMGT nomenclature committee. IMGT, International ImMunoGeneTics Information System (18). Dots represent the consensus nucleotide; abs, absent.

a *According to Edelman et al. (33)*.

b *Allotypes were inferred according to Lefranc et al. (6)*.

# *Number of copies observed in this study*.

Interestingly, some new alleles of all gene segments were observed at high frequency (*f* > 0.10; Table 5). The highest frequencies for novel alleles were observed for IGHG1^*^07 in GKW (*f* = 0.478; 34 individuals), IGHG1^*^08 in BrJAP (*f* = 0.155; 15 individuals), IGHG2^*^08 in BrJAP (*f* = 0.202; 23 individuals), IGHG2^*^09 in GRC (*f* = 0.137; 9 individuals), IGHG3^*^21 in BrJAP (*f* = 0.158; 16 individuals), and IGHG3^*^22 in GRC (*f* = 0.157; 15 individuals).

**Table 5:** One third of the novel *IGHG* alleles were observed in high frequencies (0.05 < *f* < 0.48).

		**GKW**	**GND**	**GRC**	**KIV**	**KRC**	**BrJAP**	**CTBA**
	**Sample size**	**46**	**48**	**51**	**50**	**52**	**55**	**56**
	**HW *p*-value**	**1**	**0.086**	**0.912**	**0.836**	**0.889**	**0.530**	**0.530**
IGHG1*02		0.522	0.438	0.725	0.770	0.606	0.600	0.228
IGHG1*03			0.094	0.020	0.020	0.029		0.707
IGHG1*05								0.022
IGHG1*06								0.011
IGHG1*07		0.478	0.469	0.235	0.200	0.365	0.136	0.033
IGHG1*08					0.010		0.155	
IGHG1*09							0.018	
IGHG1*10							0.018	
IGHG1*11							0.064	
IGHG1*12				0.010				
IGHG1*13							0.009	
IGHG1*14				0.010				
	**Sample size**	**46**	**48**	**51**	**52**	**52**	**57**	**47**
	**HW** ***p*****-value**	**1**	**1**	**1**	**1**	**1**	**0.232**	**0.146**
IGHG2*02			0.083	0.020	0.038	0.029	0.114	0.436
IGHG2*03		0.957	0.885	0.735	0.942	0.952	0.579	0.489
IGHG2*06								0.021
IGHG2*07		0.033				0.010		
IGHG2*08				0.010	0.019	0.010	0.202	0.032
IGHG2*09		0.011	0.010	0.137				
IGHG2*10			0.021	0.098				
IGHG2*11							0.009	
IGHG2*12							0.018	0.011
IGHG2*13							0.061	0.011
IGHG2*14							0.009	
IGHG2*15							0.009	
	**Sample size**	**46**	**48**	**51**	**52**	**51**	**57**	**51**
***Allele***	**HW** ***p*****-value**	**-**	**1**	**1**	**1**	**1**	**0.951**	**0.519**
IGHG3*01								0.020
IGHG3*10								0.010
IGHG3*11			0.094	0.010	0.038	0.020		0.588
IGHG3*12								0.069
IGHG3*14		1.000	0.844	0.833	0.952	0.971	0.474	0.186
IGHG3*15							0.009	
IGHG3*16					0.010			0.039
IGHG3*19							0.272	
IGHG3*20							0.018	
IGHG3*21							0.158	
IGHG3*22			0.063	0.157		0.010		0.029
IGHG3*23							0.009	
IGHG3*24							0.018	
IGHG3*25							0.035	
IGHG3*26								0.039
IGHG3*27								0.010
IGHG3*28								0.010
IGHG3*29							0.009	

*Novel alleles (in bold) have been confirmed by sequencing and/or molecular cloning. Their official names have been assigned by IMGT nomenclature committee. IMGT, International ImMunoGeneTics Information System (18); KIV, Kaingang from Ivaí; KRC, Kaingang from Rio das Cobras; GRC, Guarani Mbya; GKW, Guarani Kaiowa; GND, Guarani Ñandeva; BrJAP, Japanese-descendant from Curitiba; CTBA, Euro-descendants from Curitiba*.

Because most of the previous studies only described the immunoglobulin heavy chain diversity serologically, we inferred the serological Gm allotypes from our nucleotide sequence data, based on the nucleotide sequence description for each previously reported allotype (6), to allow comparison with previously reported variants. For example, the most frequent allele haplotype (alleles that are in the same chromosome and inherited together in a block) was the one comprising the gene segments IGHG1^*^02, IGHG2^*^03, IGHG3^*^14 (*f* = 0.182 to 0.740), which encodes the Gm haplotype “C” Gm21,26,27,28;17,1;(.), the most frequent lgG allotype haplotype in our populations (*f* = 0.21 to 0.77; Table 6). The correspondence between allele haplotype and allotype haplotypes are in the Table S4. More than one *IGHG* allele haplotype can define a single Gm allotype haplotype, as is the case of the Gm haplotype “B” Gm5,10,11,13,14,26,27;3;(.), that is encoded in our data by the allele haplotype IGHG3^*^11,IGHG2^*^03,IGHG2^*^03, by IGHG3^*^11,IGHG1^*^14,IGHG2^*^03, or by IGHG3^*^11,IGHG1^*^03,IGHG2^*^08. In order to simplify the interpretation of the data, Gm haplotype identifiers (from A to M) were used as suggested by Lefranc et al. (6).

**Table 6:** Gm allotype haplotypes frequencies inferred from nucleotide sequencing.

		**GKW**	**GND**	**GRC**	**KIV**	**KRC**	**BrJAP**	**CTBA**
	**Sample size**	**46**	**48**	**51**	**50**	**51**	**55**	**44**
**ID**^**a**^	**Gm haplotypes**							
A	5,10,11,13,14,26,27;3;23		0.083	0.01	0.02	0.02		0.444
B	5,10,11,13,14,26,27;3;(.)		0.01					0.277
C	21,26,27,28;17,1;(.)	0.522	0.375	0.588	0.77	0.608	0.384	0.211
D	21,26,27,28;17,1,2;(.)	0.478	0.468	0.235	0.2	0.363	0.134	0.011
I	10,11,13,15,16,27;17,1;(.)						0.286	
J	5,10,11,13,14,26,27;3,1;23				0.01		0.107	
K	5,10,11,13,14,26,27;3,1;(.)						0.045	
	21,26,27,28;3;23			0.01				
	5,10,11,13,14,26,27;17,1;23						0.009	
	10,11,13,16,27;17,1;(.)						0.009	
	21,27;17,1,2;(.)							0.022
	21,27;17,1;(.)		0.063	0.157		0.01		0.011
	5,10,11,13,14,26,27;17,1;(.)							0.022

a Allotype haplotype ID are as described by Lefranc et al. (6).

*KIV, Kaingang from Ivaí; KRC, Kaingang from Rio das Cobras; GRC, Guarani Mbya; GKW, Guarani Kaiowa; GND, Guarani Ñandeva; BrJAP, Japanese-descendants; CTBA, Euro-descendants from Curitiba*.

### Lower *IGHG* Diversity Was Observed in Amerindians and Frequencies Differed Significantly Among Populations

*IGHG* allelic frequencies varied across populations (Table 5). A small number of highly frequent alleles were observed for all gene segments in Amerindian populations. Even though Guarani populations share a more recent common ancestor, allelic frequencies significantly differed among them (*p* < 0.01), with low to moderate F_ST_ values (0.02–0.10) (Table 7). Allelic frequencies did not differ between the two Kaingang populations (*p* = 0.065; F_ST_ = 0.03). More conspicuous differences were found between the Japanese-descendant and Euro-descendant populations compared to each other, and between each of these two populations compared to the Amerindian populations, with F_ST_ values ranging from 0.11 to 0.52, indicating moderate to high genetic differentiation.

**Table 7:** Genetic differentiation for *IGHG1, IGHG2*, and *IGHG3* among populations.

	**GKW**	**GND**	**GRC**	**KIV**	**KRC**	**BrJAP**	**CTBA**
**GKW**		**	***	***	ns	***	***
**GND**	0.02828		***	***	**	***	***
**GRC**	0.10738	0.07720		***	***	***	***
**KIV**	0.10494	0.11120	0.05722		ns	***	***
**KRC**	0.01042	0.03816	0.06889	0.03220		***	***
**BrJAP**	0.21496	0.15144	0.11437	0.18492	0.19168		***
**CTBA**	0.51577	0.38142	0.41023	0.51270	0.50134	0.28576	

Upper diagonal: the statistical significance of the exact test of population differentiation between pairs of population. **p < 0.001–0.01; ***p < 0.001; ns p > 0.05. Lower diagonal: F_ST_ values between populations. Wright's F_ST_ scale: 0–0.05 (in very light gray), 0.05–0.15 (in light gray), 0.15–0.25 (in medium gray), and above 0.25 (in dark gray) indicate little, moderate, great, and very great genetic differentiation between populations. KIV, Kaingang from Ivaí; KRC, Kaingang from Rio das Cobras; GRC, Guarani Mbya; GKW, Guarani Kaiowa; GND, Guarani Ñandeva; BrJAP, Japanese-descendants; CTBA, Euro-descendants from Curitiba.

**Figure 2:**
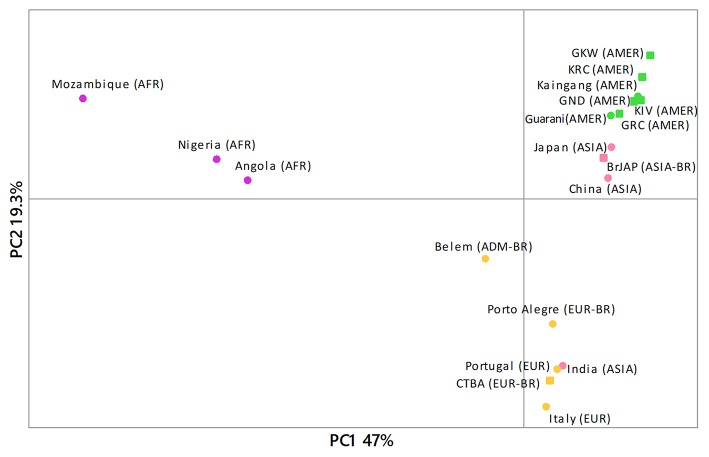
Principal component analysis using Gm allotype haplotype frequencies was consistent with geography and ancestry. For comparisons with previously described population, we inferred the Gm allotype frequencies based on the observed nucleotide sequences, according to Lefranc et al. (6). Circles represent population data from the literature and squares represent populations from the present study. All frequencies reported in the literature are listed in Table S3. AFR, African populations; AMER, Amerindian populations; ASIA, Asian populations; EUR, European populations; EUR-BR, Euro-descendant populations from Brazil; ADM-BR, Admixed population from Brazil; KIV, Kaingang from Ivaí; KRC, Kaingang from Rio das Cobras; GRC, Guarani Mbya; GKW, Guarani Kaiowa; GND, Guarani Ñandeva; BrJAP, Japanese-descendants; CTBA, Euro-descendants from Curitiba.

The principal component analysis (PCA) grouping was consistent with ancestry and geography (Figure 2). Amerindians and Asians formed two separated groups close to each other. Europeans and admixed populations of predominantly European ancestry grouped together, while Africans were more distant.

Genotypic distributions for all gene segments were in accordance with Hardy-Weinberg equilibrium in all population samples (0.08 > *p* >1).

### Distinct Linkage Disequilibrium Patterns Among Populations

Linkage disequilibrium (LD) patterns differed among populations (Figure S2). Interestingly, each Guarani population exhibited a distinct LD pattern despite their close relationship. In GKW, only five variable sites were observed in all three gene segments, of which three were in absolute LD (*D*′ = 1, *r*^2^ = 1). In contrast, more variable sites (21 and 24) were observed for the other two Guarani populations. In addition, many variants that were in LD in GND were not observed in LD in GRC.

The G1m3 allotype (*rs1071803*) and the G2m23 allotype (*rs800915*6) were in strong LD in all Amerindian populations (*D*′ = 1; *r*^2^ > 0.87), as well as in CTBA (*D*′ = 1; *r*^2^ = 0.43), and BrJAP (*D*′ = 0.73; *r*^2^ = 0.92) in which fewer SNPs were observed in strong LD.

### Sequence Analysis Suggests That Gene Conversion Between Frequent Alleles of Different Gene Segments Generated Novel Alleles

**Figure 3:**
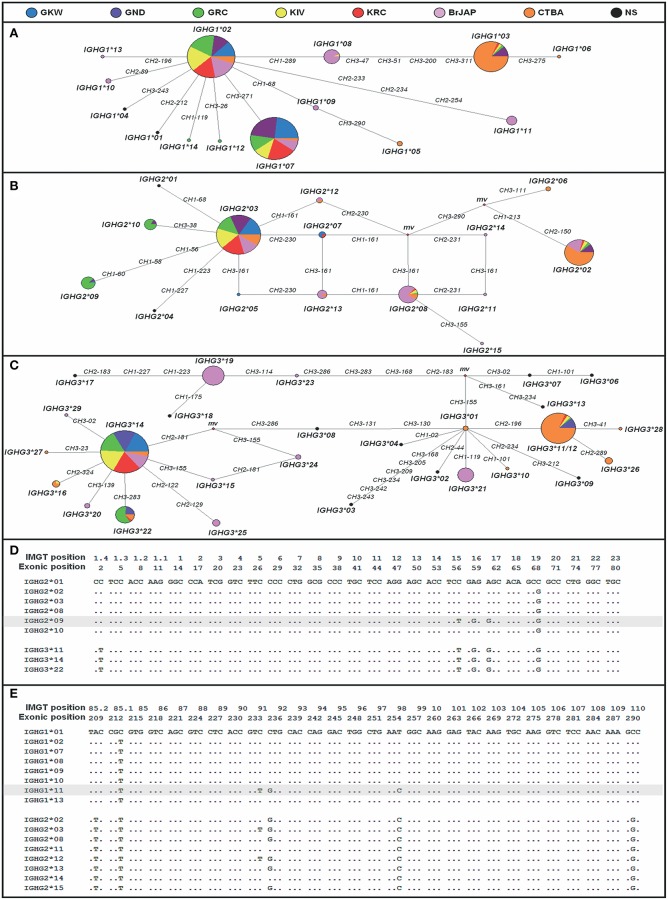
Relationship of *IGHG* alleles. Median-Joining Network of all *IGHG1* **(A)**, *IGHG2* **(B)** and *IGHG3* **(C)** alleles. Each circle (node) represents an allele and the size of each circle is proportional to the allele frequency. Numbers in the branches indicate the exon and the exonic position of nucleotide differences between alleles. The mv nodes (median vector) are possible unsampled or extinct ancestral sequences generated by the MJ algorithm to connect the alleles. Alleles IGHG3^*^11 and IGHG3^*^12 **(C)** were grouped because they do not differ in nucleotide sequence, except for the hinge size. KIV, Kaingang from Ivaí; KRC, Kaingang from Rio das Cobras; GRC, Guarani Mbya; GKW, Guarani Kaiowa; GND, Guarani Ñandeva; BrJAP, Japanese-descendants; CTBA, Euro-descendants from Curitiba; NS, not sampled (alleles not observed in this study). The occurrence of multiple mutations in the same positions in different gene segments is extremely unlikely. In addition, sequence homology and tandem positioning favor unequal crossing over between high frequent alleles. Therefore, based on the multiple alignments, we suggest that **(D)** IGHG2^*^09 allele could be a product of gene conversion between IGHG2^*^03 and IGHG3^*^14 at position 56 (T), 58 (G), and 60 (G) of CH1 exon; and **(E)** IGHG1^*^11 could be a product of gene conversion between IGHG1^*^02 and IGHG2^*^03 at position 233 (T), 234 (G), and 254 (C) in CH2 exon.

Median-Joining network (Figure 3) shows that the most frequent alleles IGHG1^*^01, IGHG2^*^03, and IGHG3^*^14 were central nodes in the network, with few nucleotides differing between them and the other alleles. The loops indicate possible recombination sites.

Alignment of all the known alleles of the *IGHG1, IGHG2*, and *IGHG3* gene segments suggests that some novel alleles discovered in this study could have been generated by gene conversion between alleles of different gene segments (Figure 3). For example, the novel allele IGHG1^*^11, present in BrJAP (*f* = 0.064), could have been generated by gene conversion between the most frequent *IGHG2* allele (IGHG2^*^03; f = 0.579) and the most frequent *IGHG1* allele (IGHG1^*^02; *f* = 0.60). In addition, gene conversion between the frequent IGHG2^*^03 and IGHG3^*^14 alleles (*f* = 0.735 and *f* = 0.833, respectively) could explain the origin of allele IGHG2^*^09 (*f* = 0.14).

### Neutrality Tests Suggest Evidence of Natural Selection Shaping *IGHG* Polymorphism

Neutrality tests performed by Tajima's *D*, Fu and Li's *D* and *F* were non-significant for most populations. However, Fay and Wu's test resulted in significant negative values for most populations, which may indicate positive selection at an adjacent site (Table 8).

**Table 8:** Fay and Wu's test was significant in the majority of the study populations.

		**GKW**	**GND**	**GRC**	**KIV**	**KRC**	**BrJAP**	**CTBA**
	**2n**	**92**	**96**	**102**	**100**	**102**	**116**	**98**
**Gene segment**	**Test**							
*IGHG1*	Tajima's D	–	–	–	–	–	–	–
	Fu and Li's D	–	–	–	–	–	–	–
	Fu and Li's F	–	–	–	–	–	–	–
	Fay and Wu's H	–	−2.455^*^	−3.400^**^	−3.384^**^	−3.490^**^	−2.36^*^	–
*IGHG2*	Tajima's D	−1.680^***^	–	–	–	−1.716^***^	–	–
	Fu and Li's D	−2.696^**^	–	–	–	–	–	–
	Fu and Li's F	−2.793^**^	–	–	–	–	–	–
	Fay and Wu's H	−1.768^**^	−2.745^*^	−4.971^**^	−5.241^***^	−5.518^***^	–	–
*IGHG3*	Tajima's D	–	–	–	–	−1.825^***^	–	–
	Fu and Li's D	–	–	–	–	–	–	−3.615^**^
	Fu and Li's F	–	–	–	–	–	–	−3.309^**^
	Fay and Wu's H	–	−4.310^**^	–	−7.253^***^	−5.670^***^	−6.307^*^	−7.372^**^

Statistical significance was tested by coalescent simulations with 10,000 repetitions:

* p < 0.01−0.05;

** p < 0.001−0.01;

*** p < 0.001;

*–p>0.05. KIV, Kaingang from Ivaí; KRC, Kaingang from Rio das Cobras; GRC, Guarani Mbya; GKW, Guarani Kaiowa; GND, Guarani Ñandeva; BrJAP, Japanese-descendants; CTBA, Euro-descendants from Curitiba*.

Deviation of neutrality was also tested by analyzing synonymous and non-synonymous substitution rates across all the known and novel alleles of all gene segments (Tables S5–S7). Overall, the rate of synonymous substitutions (dS) was significantly higher than the rate of non-synonymous (dN) substitutions (dN/dS < 1) for *IGHG1* and *IGHG2* (*p* = 0.01 and 0.032, respectively) (Table 9), consistent with purifying selection.

**Table 9:** Codon-based test indicates purifying selection shaping *IGHG1* and *IGHG2* variation.

	***IGHG1***	***IGHG2***	***IGHG3***
**Number of alleles**	**15**	**14**	**29**
Purifying selection (dN < dS)	*p* = 0.010	*p* = 0.032	ns
Positive selection (dN > dS)	ns	ns	ns

*ns, p > 0.05*.

## Discussion

Our main goal was to deliver an unprecedented and comprehensive nucleotide sequencing-based characterization of the *IGHG* gene segments in populations of different ancestries. Before this study, only 30 *IGHG* alleles have been described for *IGHG1, IGHG2*, and *IGHG3* together (18). Here, we report the discovery of 28 novel alleles, of which 16 were in a single population sample of Japanese descendants (*n* = 57) and seven in one population sample of Euro-descendants (*n* = 51). It is interesting that even in Amerindian populations, which exhibited a limited diversity, seven new alleles were found. This is clear evidence that the diversity of *IGHG* is far from being fully described and possibly a much larger number of novel alleles will be discovered as more populations are interrogated. We focused on the segments that code for the most abundant Ig in serum. Considering the homology and high sequence similarity, a different strategy would be needed for the precise characterization of *IGHG4* due to the high frequency of duplications observed for this gene segment (35).

Some of the new alleles were highly frequent. The novel allele IGHG3^*^22, frequent in Guarani Mbya (GRC, *f* = 0.157), exhibited a lower frequency in Guarani Ñandeva (GND, *f* = 0.063), and was absent in Guarani Kaiowa (GKW). These three populations share a more recent common ancestor and the differences observed can be explained by its demographic history and genetic drift. Demographic factors played a major role in shaping the diversity of other genes important for immune responses in these same Amerindian populations (36). Genetic drift, particularly founder effect and bottleneck, may explain the lower diversity of *IGHG* in Amerindians and the fluctuation of their allelic frequencies. On the other hand, the IGHG3^*^22 allele was observed only in one Kaingang individual. This fact suggests gene flow from Guarani to Kaingang. Although GRC and KRC remain isolated due to strong cultural barriers, their immediate vicinity did result in a low degree of admixture (14).

IGHG3^*^11 is the most common *IGHG3* allele in Euro-descendants (CTBA, *f* = 0.588) and was observed at lower frequency in Amerindians: GND (*f* = 0.094), GRC (*f* = 0.010), KIV (*f* = 0.038), KRC (*f* = 0.020), being absent in GKW. This allele corresponds to the allotype G3m5,10,11,13,14,26,27 which has been previously shown to be highly frequent in Europeans but absent in non-admixed Amerindians (37–42). Also, similar allele distribution was observed for IGHG1^*^03 in the study populations. These observations are consistent with previous studies from our group, which estimated the admixture rate of Guarani and Kaingang by analyzing *HLA* class II genes. In that study, the estimated admixture rate with non-Amerindians was 14.3% for GND, 3.7% for GRC, 7.2% for Kaingang, and no admixture for GKW (15). The Gm allotype haplotype frequencies inferred from DNA sequencing in our study (in which the most common haplotypes were C and D) were similar to those found in former reports that characterized serologically the Guarani and Kaingang populations from Santa Catarina State, Brazil (42), and other native American populations (41, 43, 44).

The new allele IGHG3^*^21 was frequent in BrJAP (*f* = 0.158), but absent in the other populations. According to the nucleotide sequence, it encodes the haplotype Gm5,10,11,13,14,26,27, whose frequency was previously reported as 15.2% in a study with Japanese families (45). In that same study, the haplotypes C (Gm21;17,1;(.) – 40.7%), D (Gm21;17,1,2;(.) – 16.4%), I (Gm11,13,15,16;17,1;(.) – 27.7%), and J (Gm5,11,13;3,1;23 – 15.2%) exhibited similar frequencies to the ones inferred from DNA sequencing in BrJAP, which were 38.4%, 13.4%, 28.6%, and 10.7%, respectively (Table 6). The novelty of our results is showing, for the first time, the characterization of the variants at DNA level that are responsible for the occurrence of these Gm haplotypes in Japanese populations.

Strong linkage disequilibrium (LD) (Figure S2) was observed in most Amerindian populations, as expected for these historically small populations that suffered strong genetic drift and multiple founder effects since the arrival of the first Americans to the continent and during their migration from the North to the South in the American continent. Interestingly, the patterns of LD differed among Guaranis, despite their shared ancestry. GKW exhibited a reduced number of variable sites, while GRC exhibited a reduced LD in comparison to GND. These differences could also be explained by genetic drift, as certain haplotypes that stochastically increased their frequencies in a population after their divergence may not have increased in the others.

In contrast, the Japanese-descendant and Euro-descendant populations have higher nucleotide and allele diversity and fewer SNPs in LD. Even so, SNPs from different gene segments are in LD in these urban populations. In BrJAP, SNPs of allotypes G1m17 (*rs1071803*) and G2m(.) (*rs8009156*) are in LD (*D* = 0.92; *r*^2^ = 0.73) and are present in the allotype haplotypes C and D, reported as the most common in Japanese populations (45).

In the MJ networks (Figure 3), IGHG1^*^02, IGHG2^*^03 and IGHG3^*^14 were connected with most alleles and were present at high frequencies in all populations. This pattern suggests that most of the other known alleles could have been originated from them. In the *IGHG2* MJ network, one loop shows two paths where substitutions at position 161 of exon CH3 and 230 of CH2 occurred to generate the IGHG2^*^05, ^*^07, and ^*^13 alleles. It can be hypothesized that a mutation occurred in one of them, for example, IGHG2^*^03 at position 161 of exon CH3, generated IGHG2^*^05 and this allele, likewise, might have mutated at position 230 of exon CH2 originating allele IGHG2^*^13. As independent mutations in the same positions are extremely unlikely, the fact that the IGHG2^*^07 allele has a variant in the same position (230 of CH2 exon) indicates that gene conversion between alleles IGHG2^*^13 and IGHG2^*^03 originated the IGHG2^*^07 allele. Moreover, we suggest that the novel alleles IGHG1^*^11 and IGHG2^*^09 resulted from gene conversion between two frequent alleles of different gene segments. Overall, our data point to a major role of recombination and gene conversion originating new *IGHG* alleles, which is consistent with the tandem positioning and high sequence similarity of these segments, which favor unequal crossing-over (46).

Kaingang from Ivaí and Kaingang from Rio das Cobras presented low genetic differentiation (F_ST_ = 0.032), and similar allele frequencies (Table 7), most probably because of their recent common origin and gene flow due to the absence of cultural barriers, in addition to their geographical proximity. The F_ST_ values between the Guarani populations were low to moderate, which is an evidence of genetic drift affecting the *IGHG* diversity in these populations. These results are compatible with previous reports for mtDNA in the same populations, which indicated that divergence of the three Guarani populations occurred at around 1,800 years before present (ybp), much earlier than the separation of the Kaingang populations that was estimated at of 207 ybp (13).

The PCA results (Figure 2) were consistent with geography and ancestry and showed that our data are consistent with data obtained by serologic methods, previously reported in the literature. The exception was India, which grouped with Europeans and Euro-descendants. In fact, PCA grouping does not necessarily mean common ancestry, as it can also result from migration or stochastic factors, or convergent evolution by natural selection. The grouping solely reflects the similarities of the *IGHG* allelic frequencies in these populations.

The results of most neutrality tests suggested that natural selection is not the major factor responsible for shaping *IGHG* diversity in the study populations. In other words, for *IGHG* the impact of genetic drift due to demographical processes is possibly stronger than the signal left by natural selection. As is known, Amerindians have a long history of migrations and isolation, and went through severe bottlenecks after the European colonization (14). Still, in GKW and KRC for *IGHG2* and KRC and CTBA for *IGHG3*, the results of Tajima's *D*, and Fu and Li's *D* and *F* tests indicated diversity sweeps due to bottlenecks or purifying selection.

Analyzing all the currently known *IGHG* alleles, including the 28 novel alleles that we here described, we found that the codon-based dN/dS test showed significant results for purifying selection (Table 9) for *IGHG1* (*p* = 0.01) and *IGHG2* (*p* = 0.03). We observed that synonymous (dS) substitution rates were higher than non-synonymous (dN) substitution rates. It was previously demonstrated that Gm1 allotypes have a different impact on the IgG1 ability to bind the Fc gamma receptor (FcγR)-like proteins from viruses. Antibodies with G1m1,2,17 allotype exhibit lower affinity to the viral FcγR-like protein of the human cytomegalovirus (HCMV), which decreases susceptibility to this infection (47). Similarly, the FcγR-like protein from herpes simplex virus (HSV) binds with lower affinity to antibodies carrying the G1m3 allotype due to certain residues in the CH1 and CH3 domains (9). In the light of our results, it is plausible to suggest that emerging amino acid replacements that favored binding to viral proteins were negatively selected as a result of their deleterious effect for the individuals carrying the mutations. Higher binding to these viral proteins would favor viral evasion from immune responses and increase the susceptibility to certain viral infections. Moreover, purifying selection against non-synonymous changes could have limited the diversification of *IGHG1* and *IGHG2*.

The Fay and Wu *H* test was significant with negative values for almost every population and gene segment analyzed. This could be interpreted as a result of an excess of derived variants at high frequencies in the gene genealogies. Fay and Wu (30) suggested that this may be a unique pattern produced by hitchhiking of variants in the vicinity that are being favored by positive selection. *IGHG* gene segments are located downstream of the *IGHV, IGHD*, and *IGHJ* gene segments that encode the immunoglobulin variable regions, which specifically bind to antigens (2, 4). Therefore, we suggest that selection for variants in the variable region may be impacting the diversity of the constant region by hitchhiking mutations in the *IGHG* gene segments. This hypothesis is corroborated by the findings of Tanaka and Nei (48), who demonstrated that the non-synonymous mutation rate was higher than the synonymous rate in the gene segments that code for the Ig variable region. Their results were consistent with diversity-enhancing selection or overdominant selection driving the nucleotide diversity in the variable region.

## Conclusion

Antibodies are pivotal for human survival, at both the individual and the population levels. It is surprising that despite decades of compelling evidence about the importance of the immunoglobulin gene variation for human immunity and the not so recent advent of sequencing technologies, most of the knowledge about *IGHG* is still based on serologic typing. As we see here, the fact that the regions encoded by *IGHG* are called “constant” does not mean these segments are not highly polymorphic. In fact, we found 16 novel alleles in a population sample of only 57 Japanese descendants. The *IGHG* genomic region is not well-covered in genome-wide association studies and whole genome sequencing databases. The homology and high sequence similarity of *IGHG* segments impose technical difficulties for sequencing, particularly at large scale. Besides, the somatic recombination events characteristic of the *IGH* locus makes DNA from B-cell lines, used in so many studies, not suitable for *IGHG* sequencing. Our study is the first to sequence systematically these segments at the nucleotide level in populations. We here present a full characterization of *IGHG1-3* diversity in seven Brazilian populations, linkage disequilibrium, haplotypes and evidence of purifying selection and genetic drift. Understanding the *IGHG* normal variation in populations and its evolution may be the key to better comprehend how the immune system fights invading organisms and non-self-antigens and also may contribute to the development of new vaccines.

## Ethics Statement

This study was carried out in accordance with the recommendations of Brazilian National Human Research Ethics Committee (CONEP) with written informed consent from all subjects. All subjects gave written informed consent in accordance with the Declaration of Helsinki. The protocol was approved by the Brazilian National Human Research Ethics Committee (CONEP).

## Author Contributions

DA designed the study. VC-S, DA, LV, RD, and HI performed DNA sequencing and genotyping. VC-S analyzed the data. RW, VC-S, RD, HI, and LV performed molecular cloning and validation of novel alleles. MP-E, DA, DM, and RW contributed with reagents. VC-S, DA, MP-E, DM, and MB drafted the manuscript. All authors significantly contributed with ideas and critically reviewed this manuscript.

### Conflict of Interest Statement

The authors declare that the research was conducted in the absence of any commercial or financial relationships that could be construed as a potential conflict of interest.

## Supplementary Material

Figure S1 Location of IGHG1, IGHG2, and IGHG3 primers. Arrows indicate primers and their direction. All primer sequences are listed in Table S2. Primers used for amplification and sequencing are shown in blue, green, and red; primers only used for sequencing are represented in gray. This representation is not to scale.

Figure S2 Linkage disequilibrium among SNPs within *IGHG3, IGHG1*, and *IGHG2*. The maximum values of D'/ LOD are represented in red and the minimum values are in gray and white. Within each rhomb are represented values of *r*^2^ (×100); when it is empty (no values shown) it indicates *r*^2^ = 100 (absolute linkage). KIV, Kaingang from Ivaí; KRC, Kaingang from Rio das Cobras; GRC, Guarani Mbya; GKW, Guarani Kaiowa; GND, Guarani Ñandeva; BrJAP, Japanese-descendants; CTBA, Euro-descendants from Curitiba.

Table S1 The seven population samples analyzed in this study.

Table S2 Description of amplification and sequencing primers.

Table S3 Allotype haplotype frequencies of populations from various continents available in the literature.

Table S4 Correspondence between haplotypes of *IGHG* alleles ad haplotypes of Gm allotypes and their observed frequencies.

Table S5 Statistical significance of codon-based test of selection between sequences of *IGHG1* gene segment.

Table S6 Statistical significance of codon-based test of selection between sequences of *IGHG2* gene segment.

Table S7 Statistical significance of codon-based test of selection between sequences of *IGHG3* gene segment.

## References

[1] Schroeder HW Cavacini L. Structure and function of immunoglobulins. J Allergy Clin Immunol. (2010) 125:S41–52. 10.1016/j.jaci.2009.09.046 20176268 PMC3670108

[2] Beale D Feinstein A. Structure and function of the constant regions of immunoglobulins. Q Rev Biophys. (1976) 9:135. 10.1017/S0033583500002390 785525

[3] Croce CM Shander M Martinis J Cicurel L D'Ancona GG Dolby TW. Chromosomal location of the genes for human immunoglobulin heavy chains. Proc Natl Acad Sci USA. (1979) 76:3416–9. 10.1073/pnas.76.7.3416 114999 PMC383836

[4] McBride OW Battey J Hollis GF Swan DC Siebenlist U Leder P. Localization of human variable and constant region immunoglobulin heavy chain genes on subtelomeric band q32 of chromosome 14. Nucleic Acids Res. (1982) 10:8155–70. 10.1093/nar/10.24.8155 6819544 PMC327076

[5] Lefranc M-P Lefranc G The Immunoglobulin FactsBook. London; San Diego, CA: Academic Press (2001).

[6] Lefranc M-P Lefranc G. Human Gm, Km, and Am allotypes and their molecular characterization: a remarkable demonstration of polymorphism. Methods Mol Biol. (2012) 882:635–80. 10.1007/978-1-61779-842-9_34 22665258

[7] Granoff DM Holmes SJ. G2m(23) immunoglobulin allotype and immunity to *Haemophilus influenzae* type b. J Infect Dis. (1992) 165(Suppl. 1):S66–9. 10.1093/infdis/165-Supplement_1-S66 1588180

[8] Pandey JP Kistner-Griffin E Iwasaki M Bu S Deepe R Black L. Genetic markers of immunoglobulin G and susceptibility to breast cancer. Hum Immunol. (2012) 73:1155–8. 10.1016/j.humimm.2012.07.340 22884983

[9] Atherton A Armour KL Bell S Minson AC Clark MR. The herpes simplex virus type 1 Fc receptor discriminates between IgG1 allotypes. Eur J Immunol. (2000) 30:2540–7. 10.1002/1521-4141(200009)30:9&lt;2540::AID-IMMU2540&gt;3.0.CO;2-S 11009087

[10] Dard P Lefranc MP Osipova L Sanchez-Mazas A. DNA sequence variability of IGHG3 alleles associated to the main G3m haplotypes in human populations. Eur J Hum Genet. (2001) 9:765–72. 10.1038/sj.ejhg.5200700 11781688

[11] Huck S Fort P Crawford DH Lefranc MP Lefranc G Sequence of a human immunoglobulin gamma 3 heavy chain constant region gene: comparison with the other human Cγ genes. Nucleic Acids Res. (1986) 14:1779–89. 10.1093/nar/14.4.1779 3081877 PMC339572

[12] Pandey JP Li Z. The forgotten tale of immunoglobulin allotypes in cancer risk and treatment. Exp Hematol Oncol. (2013) 2:6. 10.1186/2162-3619-2-6 23425356 PMC3598368

[13] Marrero AR Silva-Junior WA Bravi CM Hutz MH Petzl-Erler ML Ruiz-Linares A. Demographic and evolutionary trajectories of the Guarani and Kaingang natives of Brazil. Am J Phys Anthropol. (2007) 132:301–10. 10.1002/ajpa.20515 17133437

[14] Petzl-Erler ML Luz R Sotomaior VS. The HLA polymorphism of two distinctive South-American Indian tribes: the Kaingang and the Guarani. Tissue Antigens. (1993) 41:227–37. 10.1111/j.1399-0039.1993.tb02011.x 8236235

[15] Tsuneto LT Probst CM Hutz MH Salzano FM Rodriguez-Delfin LA Zago MA HLA class II diversity in seven Amerindian populations. Clues about the origins of the Ach?? Tissue Antigens. (2003) 62:512–26. 10.1046/j.1399-0039.2003.00139.x 14617035

[16] Lahiri DK Nurnberger JI. A rapid non-enzymatic method for the preparation of HMW DNA from blood for RFLP studies. Nucleic Acids Res. (1991) 19:5444. 10.1093/nar/19.19.5444 1681511 PMC328920

[17] Sambrook J Fritsch EF Maniatis T Molecular Cloning: A Laboratory Manual. Cold Spring Harbor, NY: Cold Spring Harb Lab Press (1989).

[18] Lefranc M-P Giudicelli V Duroux P Jabado-Michaloud J Folch G Aouinti S. IMGT®, the international ImMunoGeneTics information system® 25 years on. Nucleic Acids Res. (2015) 43:D413–22. 10.1093/nar/gku1056 25378316 PMC4383898

[19] Peakall R Smouse PE GENALEX 6: genetic analysis in Excel. Population genetic software for teaching and research. Mol Ecol Notes. (2006) 6:288–95. 10.1111/j.1471-8286.2005.01155.x PMC3463245 22820204

[20] Guo S Thompson E. Performing the exact test of Hardy–Weinberg proportion for multiple alleles. Biometrics. (1992) 48:361–72. 10.2307/2532296 1637966

[21] Excoffier L Lischer HEL. Arlequin suite ver 3.5: a new series of programs to perform population genetics analyses under Linux and Windows. Mol Ecol Resour. (2010) 10:564–67. 10.1111/j.1755-0998.2010.02847.x 21565059

[22] Barrett JC Fry B Maller J Daly MJ. Haploview: analysis and visualization of LD and haplotype maps. Bioinformatics. (2005) 21:263–5. 10.1093/bioinformatics/bth457 15297300

[23] Bandelt HJ Forster P Rohl A. Median-joining networks for inferring intraspecific phylogenies. Mol Biol Evol. (1999) 16:37–48. 10.1093/oxfordjournals.molbev.a026036 10331250

[24] Raymond M Rousset F. An exact test for population differenciation. Evolution. (1995) 49:1280–3. 10.1111/j.1558-5646.1995.tb04456.x 28568523

[25] Reynolds J Weir BS Cockerham CC. Estimation of the coancestry coefficient: basis for a short-term genetic distance. Genetics. (1983) 105:767–79. 17246175 10.1093/genetics/105.3.767 PMC1202185

[26] Slatkin M. A measure of population subdivision based on microsatellite allele frequencies. Genetics. (1995) 139:457–62. 7705646 10.1093/genetics/139.1.457 PMC1206343

[27] Minitab Inc Minitab 17 Statistical Software. (2010) Available online at: www.minitab.com (accessed November 30, 2016).

[28] Tajima F. Statistical method for testing the neutral mutation hypothesis by DNA polymorphism. Genetics. (1989) 123:585–95. 2513255 10.1093/genetics/123.3.585 PMC1203831

[29] Fu YX Li WH. Statistical tests of neutrality of mutations. Genetics. (1993) 133:693–709. 8454210 10.1093/genetics/133.3.693 PMC1205353

[30] Fay JC Wu CI. Hitchhiking under positive Darwinian selection. Genetics. (2000) 155:1405–13. Available online at: https://www.ncbi.nlm.nih.gov/pubmed/10880498 10880498 10.1093/genetics/155.3.1405 PMC1461156

[31] Librado P Rozas J. DnaSP v5: a software for comprehensive analysis of DNA polymorphism data. Bioinformatics. (2009) 25:1451–2. 10.1093/bioinformatics/btp187 19346325

[32] Grantham R. Amino acid difference formula to help explain protein evolution. Science. (1974) 185:862–4. 10.1126/science.185.4154.862 4843792

[33] Edelman GM Cunningham BA Gall WE Gottlieb PD Rutishauser U Waxdal MJ. The covalent structure of an entire gammaG immunoglobulin molecule. Proc Natl Acad Sci USA. (1969) 63:78–85. 10.1073/pnas.63.1.78 5257969 PMC534037

[34] Sherry ST Ward M Kholodov M Baker J Phan L Smigielski EM. dbSNP : the NCBI database of genetic variation. Nucleic Acids Res. (2001) 29:308–11. 10.1093/nar/29.1.308 11125122 PMC29783

[35] Brusco A Cinque F Saviozzi S Boccazzi C DeMarchi M Carbonara AO. The G4 gene is duplicated in 44% of human immunoglobulin heavy chain constant region haplotypes. Hum Genet. (1997) 100:84–9. 10.1007/s004390050470 9225974

[36] Augusto DG Piovezan BZ Tsuneto LT Callegari-Jacques SM Petzl-Erler ML. KIR gene content in amerindians indicates influence of demographic factors. PLoS ONE. (2013) 8:e56755. 10.1371/journal.pone.0056755 23451080 PMC3581531

[37] Schanfield MS Gergely J Fudenberg HH Immunoglobulin allotypes of European populations. Hum Hered. (1975) 25:370–7. 10.1159/000152748 1222944

[38] Fortes-Lima C Dugoujon JM Hernández CL Reales G Calderón R. Immunoglobulin genes in andalusia (Spain). Genetic diversity in the mediterranean space. C R Biol. (2014) 337:646–56. 10.1016/j.crvi.2014.08.004 25444709

[39] Piazza A van Loghem E de Lange G Curtoni ES Ulizzi L Terrenato L. Immunoglobulin allotypes in Sardinia. Am J Hum Genet. (1976) 28:77–86. 1247022 PMC1684902

[40] Pandey JP Shannon BT Arala-Chaves MP Fudenberg HH. Gm and Km frequencies in a Portuguese population. Hum Genet. (1982) 61:154–6. 10.1007/BF00274207 6957377

[41] Lúcia M Hamel H Salzano FM Freitas MJM The Gm polymorphism and racial admixture in six Amazonian populations. J Hum Evol. (1984) 13:517–29. 10.1016/S0047-2484(84)80005-6

[42] Salzano FM Steinberg AG. The Gm and Inv groups of Indians from Santa Catarina, Brazil. Am J Hum Genet. (1965) 17:273–9. 14295495 PMC1932608

[43] Johnson WE Kohn PH Steinberg AG Population genetics of the human allotypes Gm, Inv, and A2m: an analytical review. Clin Immunol Immunopathol. (1977) 7:97–113. 10.1016/0090-1229(77)90034-4 404106

[44] Schanfield MS. Immunoglobulin allotypes (GM and KM) indicate multiple founding populations of Native Americans: evidence of at least four migrations to the New World. Hum Biol. (1992) 64:381–402. 1607185

[45] Van Loghem E Natvig JB Matsumoto H Genetic markers of immunoglobulins in Japanese families Inheritance of associated markers belonging to one IgA and three IgG subclasses. Ann Hum Genet. (1970) 33:351–9. 10.1111/j.1469-1809.1970.tb01661.x

[46] Chen J-M Cooper DN Chuzhanova N Férec C Patrinos GP. Gene conversion: mechanisms, evolution and human disease. Nat Rev Genet. (2007) 8:762–75. 10.1038/nrg2193 17846636

[47] Namboodiri AM Pandey JP. The human cytomegalovirus TRL11/IRL11-encoded FcγR binds differentially to allelic variants of immunoglobulin G1. Arch Virol. (2011) 156:907–10. 10.1007/s00705-011-0937-8 21311920

[48] Tanaka T Nei M. Positive darwinian selection observed at the variable-region genes of immunoglobulins. Mol Biol Evol. (1989) 6:447–59. 2796726 10.1093/oxfordjournals.molbev.a040569

