# Supplementary figures and images for: Unveiling the Diversity of Immunoglobulin Heavy Constant Gamma (IGHG) Gene Segments in Brazilian Populations Reveals 28 Novel Alleles and Evidence of Gene Conversion and Natural Selection
Source: Front Immunol. 2019 Jun 4;10:1161. doi: 10.3389/fimmu.2019.01161 (PMC6558194; doi:10.3389/fimmu.2019.01161)

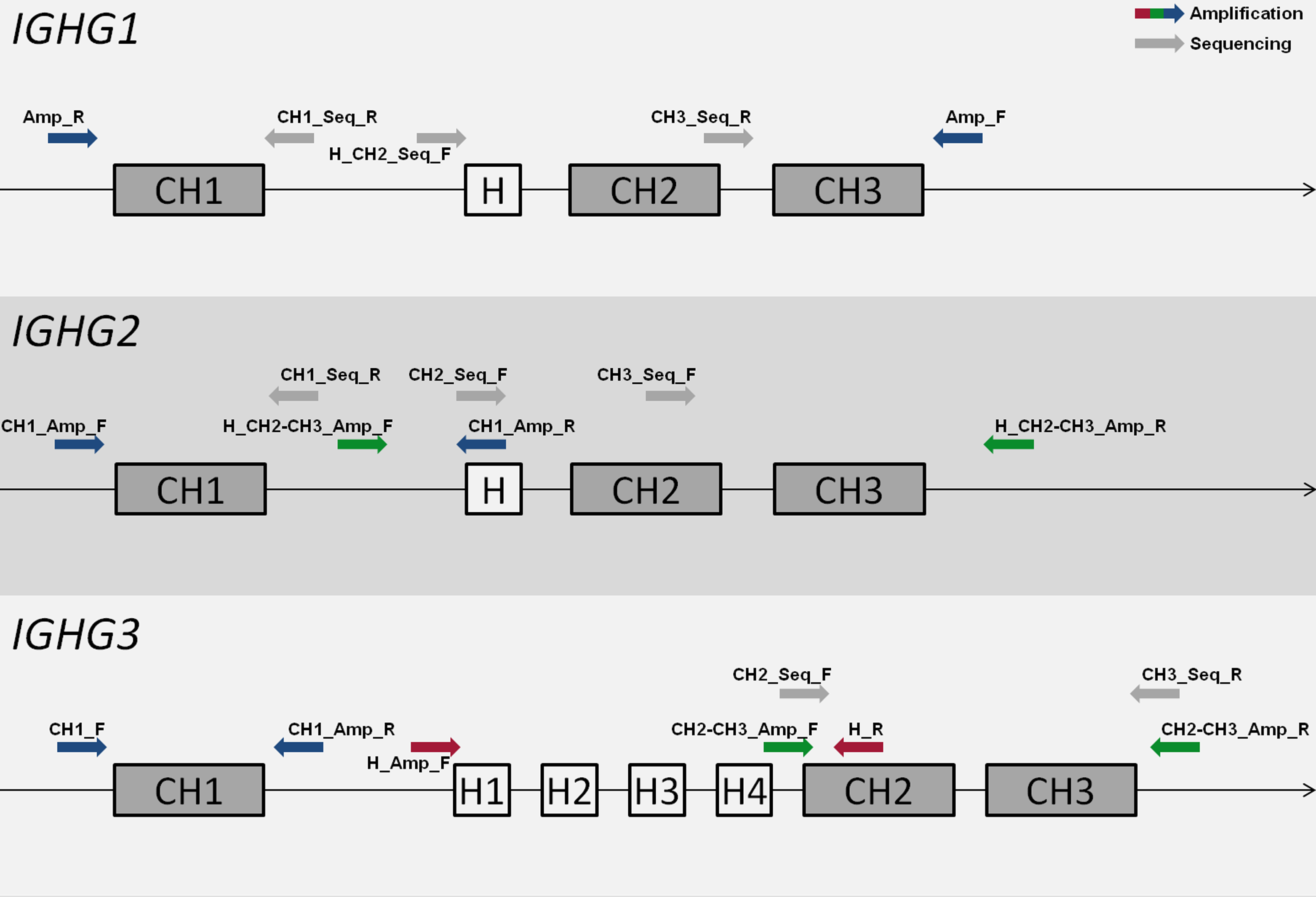

Supplement: Figure S1 — Location of IGHG1, IGHG2, and IGHG3 primers. Arrows indicate primers and their direction. All primer sequences are listed in Table S2. Primers used for amplification and sequencing are shown in blue, green, and red; primers only used for sequencing are represented in gray. This representation is not to scale. [file Image_1.TIF]

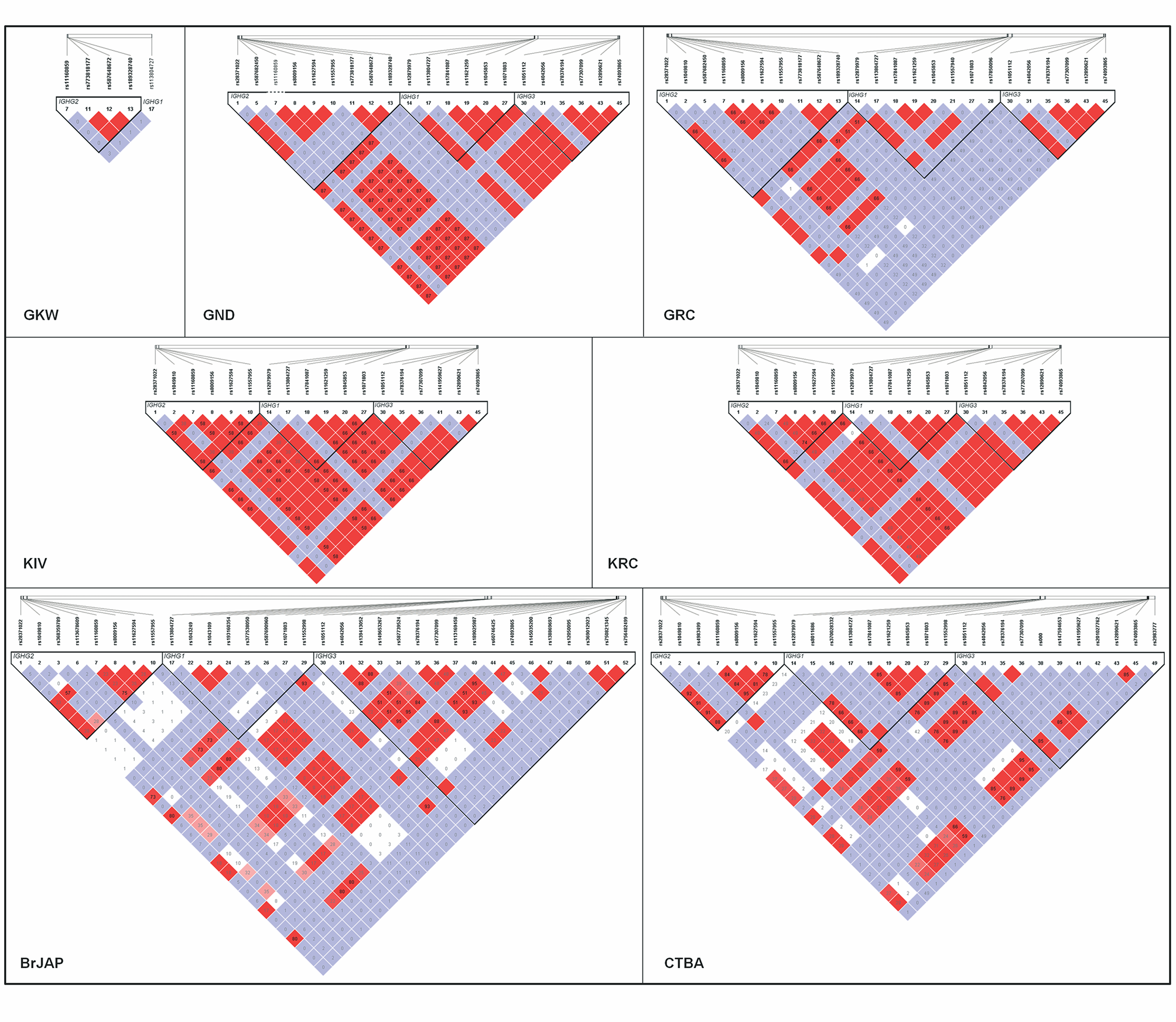

Supplement: Figure S2 — Linkage disequilibrium among SNPs within IGHG3, IGHG1, and IGHG2. The maximum values of D'/ LOD are represented in red and the minimum values are in gray and white. Within each rhomb are represented values of r2 (×100); when it is empty (no values shown) it indicates r2 = 100 (absolute linkage). KIV, Kaingang from Ivaí; KRC, Kaingang from Rio das Cobras; GRC, Guarani Mbya; GKW, Guarani Kaiowa; GND, Guarani Ñandeva; BrJAP, Japanese-descendants; CTBA, Euro-descendants from Curitiba. [file Image_2.TIF]
