# Supplementary material for: Unveiling the Diversity of Immunoglobulin Heavy Constant Gamma (IGHG) Gene Segments in Brazilian Populations Reveals 28 Novel Alleles and Evidence of Gene Conversion and Natural Selection
Source: Front Immunol. 2019 Jun 4;10:1161. doi: 10.3389/fimmu.2019.01161 (PMC6558194; doi:10.3389/fimmu.2019.01161)
Supplement: Table S1 — The seven population samples analyzed in this study. [file Table_1.docx]

**Supplementary Material**

Table S1: The seven population samples analyzed in this study

| **Population** | **Abbreviation** | **Sample Size** | **Population location** | **Geographic coordinate** |
| --- | --- | --- | --- | --- |
| Guarani Kaiowá | GKW | 46 | Amambai - MS  Limão Verde - MS | 23°06’ S, 55°12’ W  23°12’ S, 55°06’ W |
| Guarani Ñandeva | GND | 48 | Amambai - MS  Porto Lindo - MS | 23°06’ S, 55°12’ W  23°48’ S, 54°30’ W |
| Guarani Mbya | GRC | 51 | Rio das Cobras - PR | 25°18’ S, 52°32’ W |
| Kaingang from Ivaí | KIV | 52 | Ivaí-PR | 24°30’S, 51°40’W |
| Kaingang from Rio das Cobras | KRC | 52 | Rio das Cobras - PR | 25°18’ S, 52°32’ W |
| Japanese-descendant | BrJAP | 57 | Curitiba - PR | 25°25′ S, 49°17′ W |
| Euro-descendant | CTBA | 51 | Curitiba- PR | 25°25′ S, 49°17′ W |

MS: Mato Grosso do Sul State (Brazil); PR: Paraná State (Brazil)

Table S2: Description of amplification and sequencing primers

| **Gene segment** | **Exons** | **Amplification primer**  **name** | **Primer location*** | **Primer sequence 5’**🡪**3’** | **TmA(ºC)** | **TmB(ºC)** | **Exon** | **Sequencing primer**  **name** | **Primer location*** | **Primer sequence 5’**🡪**3’** |
| --- | --- | --- | --- | --- | --- | --- | --- | --- | --- | --- |
| *IGHG1* | CH1;H;CH2;CH3 | *IGHG1*_Amp_R | 106209502-106209484 | CTGAACCTCGCGGACAGTT | 64 | 60 | CH1 | *IGHG1*_CH1_Seq_R | 106208907-106208890 | CCTGGGTTAGGGGCACCT |
|  |  | *IGHG1*_Amp_F | 106207677-106207657 | GAAAGAACCATCACAGTCTCG |  |  | CH2 | *IGHG1*_H_CH2_Seq_F | 106208772-106208751 | ACACCTTCTCTCCTCCCAGATT |
|  |  |  |  |  |  |  | CH3 | *IGHG1*_CH3_Seq_F | 106208235-106208219 | CCAAAGGTGGGACCCGT |
| *IGHG2* | CH1 | *IGHG2*_CH1_Amp_F | 106111230-106111215 | GACCCTGCCTGGACCC | 64 | 60 | CH1 | *IGHG2*_CH1_Seq_R | 106110819-106110802 | GACACCCTCCCTCCCTGA |
|  |  | *IGHG2*_CH1_Amp_R | 106110437-106110418 | GCACTCGACACAACATTTGC |  |  |  |  |  |  |
|  | H;CH2;CH3 | *IGHG2*_H_CH2-CH3_Amp_F | 106110525-106110509 | CCCCAAAGGCCAAACTG | 64 | 60 | H | *IGHG2*_H_CH2-CH3_Amp_F |  |  |
|  |  | *IGHG2*_H_CH2-CH3_Amp_R | 106109374-106109356 | CACTCAGGCCTCAGACTCG |  |  | CH2 | *IGHG2*_CH2_Seq_F | 106110440-106110423 | AGCGCAAATGTTGTGTCG |
|  |  |  |  |  |  |  | CH3 | *IGHG2*_CH3_Seq_F | 106110072-106110056 | CAGCGTCCTCACCGTYG |
| *IGHG3* | CH1 | *IGHG3*_CH1_F | 106237786-106237769 | GCTCTGTCCCACACCGCA | 67 | 65 | CH1 | *IGHG3*_CH1_F |  |  |
|  |  | *IGHG3*_CH1_Amp_R | 106237170-106237154 | AGGGGCAGGGTCCTCCT |  |  |  |  |  |  |
|  | H | *IGHG3*_H_Amp_F | 106237139-106237117 | CCAAAGGCCAAACTCTCTACTCA | 65 | 63 | H | *IGHG3*_H_R |  |  |
|  |  | *IGHG3*_H_R | 106236268-106236247 | GGTCCGGGAAATCATAAGGGTA |  |  |  |  |  |  |
|  | CH2;CH3 | *IGHG3*_CH2-CH3_Amp_F | 106236405-106236386 | AGGACAGGTGCCCTAGAGTG | 64 | 60 | CH2 | *IGHG3*_CH2_Seq_F | 106236383-106236366 | CTGCATCCAGGGACAGGT |
|  |  | *IGHG3*_CH2-CH3_Amp_R | 106235527-106235511 | CGTGCCAAGCATCCTCG |  |  | CH3 | *IGHG3*_CH3_Seq_R | 106235535-106235521 | ATCCTCGCGCGACCC |

TmA(ºC) is the temperature of primer annealing in the first 10 cycles of the amplification reaction and TmB(ºC) is the temperature in the following 25 cycles.

*Coordinate at chromosome 14 location (GRCh37.p13 primary assembly)

Table S3: Allotype haplotype frequencies of populations from various continents available in the literature

| Population | Continent | Sample size | A-B | C | D | E | F | G | H | I | J-K | Reference |
| --- | --- | --- | --- | --- | --- | --- | --- | --- | --- | --- | --- | --- |
| Kaingang | AMER | 52 |  | 0.69 | 0.26 |  |  |  |  |  | 0.05 | (Salzano; Steinberg, 1965) |
| Guarani | AMER | 34 |  | 0.72 | 0.22 |  |  |  |  |  | 0.06 | (Salzano; Steinberg, 1965) |
| Japan | ASIA | 212 |  | 0.407 | 0.164 |  |  |  |  | 0.277 | 0.152 | (Loghem; Natvig; Matsumoto, 1970) |
| China | ASIA | 195 | 0.241 | 0.428 | 0.214 |  |  |  |  | 0.116 |  | (Matsumoto et al., 1986) |
| India | ASIA | 124 | 0.297 | 0.182 | 0.018 |  |  |  |  |  | 0.503 | (Vos; Kirk; Steinberg, 1963) |
| Italy | EUR | 86 | 0.831 | 0.12 | 0.049 |  |  |  |  |  |  | (Piazza et al., 1976) |
| Portugal | EUR | 78 | 0.756 | 0.111 | 0.133 |  |  |  |  |  |  | (Pandey et al., 1982) |
| Porto Alegre | EUR-BR | 163 | 0.58 | 0.28 | 0.06 | 0.08 |  |  |  |  |  | (Schneider; Salzano, 1979) |
| Belem | ADM-BR | 515 | 0.37 | 0.19 | 0.12 | 0.3 | 0.02 |  |  |  |  | (Schneider; Salzano, 1979) |
| Nigeria | AFR | 214 |  |  |  | 0.678 | 0.061 | 0.203 | 0 | 0.051 |  | (Loghem et al., 1978) |
| Angola | AFR | 111 |  |  |  | 0.793 | 0.043 | 0.164 | 0 |  |  | (Jenkins; Zoutendyk; Steinberg, 1970) |
| Mozambique | AFR | 119 |  |  |  | 0.716 | 0.076 | 0.185 | 0.024 |  |  | (Jenkins; Zoutendyk; Steinberg, 1970) |

The classification of the allotypes haplotypes (A-K) were inferred in accordance to the described in Lefranc and Lefranc (2012) (LEFRANC; LEFRANC, 2012), and based on the description of allotypes haplotypes found in the literature mentioned. Sample size represents the number of individuals tested.

References:

Jenkins, T.; Zoutendyk, A.; Steinberg, A. G. Gammaglobulin groups (Gm and Inv) of various Southern African populations. American Journal of Physical Anthropology, v. 32, n. 2, p. 197–218, 1970.

Lefranc, M. P.; Lefranc, G. Human Gm, Km, and Am allotypes and their molecular characterization: A remarkable demonstration of polymorphism. Methods in Molecular Biology, v. 882, p. 635–680, 2012.

Loghem, E. et al. Immunoglobulin allotypes in african populations: Gm-Am haplotypes in a Nigerian population. European Journal of Immunogenetics, v. 5, n. 3, p. 143–147, jun. 1978.

Loghem, E. Van; Natvig, J. B.; Matsumoto, H. Genetic markers of immunoglobulins in Japanese families Inheritance of associated markers belonging to one IgA and three IgG subclasses. Annals of Human Genetics, v. 33, n. 4, p. 351–359,1970.

Matsumoto, H. et al. Distribution of Gm and Km allotypes among five populations in China. Am J Phys Anthropol, v. 70, n. 2, p. 161–165, 1986.

Pandey, J. P. et al. Gm and Km frequencies in a Portuguese population. Human Genetics, v. 61, n. 2, p. 154–156, 1982.

Piazza, A. et al. Immunoglobulin allotypes in Sardinia. Am J Hum Genet, v. 28, n. 1, p. 77–86, 1976.

Salzano, F. M.; Steinberg, A. G. The Gm and Inv groups of Indians from Santa Catarina, Brazil. American journal of human genetics, v. 17, n. 3, p. 273–9, 1965.

Schneider, H.; Salzano, F. M. Gm allotypes and racial admixture in two Brazilian populations. Human Genetics, v. 53, n. 1, p. 101–105, 1979.

Vos, G. H.; Kirk, R. L.; Steinberg, A. G. The distribution of the gamma globulin types Gm(a), Gm(x) and Gm-like in South and Southeast Asia and Australia. Am J Hum Genet, v. 15, n. x, p. 44–52, 1963.

Table S4: Correspondence between haplotypes of *IGHG* alleles ad haplotypes of Gm allotypes and their observed frequencies

|  |  |  |  |  | **GKW** | **GND** | **GRC** | **KIV** | **KRC** | **BrJAP** | **CTBA** |
| --- | --- | --- | --- | --- | --- | --- | --- | --- | --- | --- | --- |
|  |  |  |  | **2n=** | **92** | **96** | **102** | **100** | **102** | **110** | **88** |
| **ID^1^** | **Gm allotype haplotype** | ***IGHG3*** | ***IGHG1*** | ***IGHG2*** |  |  |  |  |  |  |  |
| A | 5,10,11,13,14,26,27;3;23 | *11 | *03 | *02 |  | 0.083 | 0.010 | 0.020 | 0.020 |  | 0.394 |
| A | 5,10,11,13,14,26,27;3;23 | *12 | *03 | *02 |  |  |  |  |  |  | 0.037 |
| A | 5,10,11,13,14,26,27;3;23 | *26 | *03 | *02 |  |  |  |  |  |  | 0.023 |
| B | 5,10,11,13,14,26,27;3;(..) | *11 | *03 | *03 |  | 0.010 |  |  |  |  | 0.175 |
| B | 5,10,11,13,14,26,27;3;(..) | *11 | *06 | *03 |  |  |  |  |  |  | 0.011 |
| B | 5,10,11,13,14,26,27;3;(..) | *11 | *03 | *08 |  |  |  |  |  |  | 0.011 |
| B | 5,10,11,13,14,26,27;3;(..) | *11 | *03 | *13 |  |  |  |  |  |  | 0.011 |
| B | 5,10,11,13,14,26,27;3;(..) | *12 | *03 | *03 |  |  |  |  |  |  | 0.042 |
| B | 5,10,11,13,14,26,27;3;(..) | *28 | *03 | *03 |  |  |  |  |  |  | 0.011 |
| C | 21,26,27,28;17,1;(..) | *14 | *02 | *03 | 0.489 | 0.354 | 0.490 | 0.740 | 0.598 | 0.282 | 0.182 |
| C | 21,26,27,28;17,1;(..) | *14 | *02 | *07 | 0.033 |  |  |  | 0.010 |  |  |
| C | 21,26,27,28;17,1;(..) | *14 | *02 | *10 |  | 0.021 | 0.088 |  |  |  |  |
| C | 21,26,27,28;17,1;(..) | *14 | *12 | *03 |  |  | 0.010 |  |  |  |  |
| C | 21,26,27,28;17,1;(..) | *16 | *02 | *03 |  |  |  | 0.010 |  |  | 0.034 |
| C | 21,26,27,28;17,1;(..) | *14 | *02 | *08 |  |  |  | 0.020 |  | 0.027 |  |
| C | 21,26,27,28;17,1;(..) | *14 | *10 | *08 |  |  |  |  |  | 0.018 |  |
| C | 21,26,27,28;17,1;(..) | *20 | *02 | *03 |  |  |  |  |  | 0.018 |  |
| C | 21,26,27,28;17,1;(..) | *15 | *02 | *03 |  |  |  |  |  | 0.009 |  |
| C | 21,26,27,28;17,1;(..) | *14 | *09 | *03 |  |  |  |  |  | 0.018 |  |
| C | 21,26,27,28;17,1;(..) | *25 | *02 | *03 |  |  |  |  |  | 0.027 |  |
| C | 21,26,27,28;17,1;(..) | *25 | *02 | *12 |  |  |  |  |  | 0.009 |  |
| C | 21,26,27,28;17,1;(..) | *14 | *02 | *13 |  |  |  |  |  | 0.009 |  |
| D | 21,26,27,28;17,1,2;(..) | *14 | *07 | *03 | 0.467 | 0.458 | 0.098 | 0.200 | 0.353 | 0.118 |  |
| D | 21,26,27,28;17,1,2;(..) | *14 | *07 | *09 | 0.011 | 0.010 | 0.137 |  |  |  |  |
| D | 21,26,27,28;17,1,2;(..) | *14 | *07 | *08 |  |  |  |  | 0.010 |  | 0.011 |
| D | 21,26,27,28;17,1,2;(..) | *14 | *07 | *15 |  |  |  |  |  | 0.009 |  |
| D | 21,26,27,28;17,1,2;(..) | *14 | *07 | *14 |  |  |  |  |  | 0.009 |  |
| I | 10,11,13,15,16,27;17,1;(..) | *19 | *02 | *08 |  |  |  |  |  | 0.127 |  |
| I | 10,11,13,15,16,27;17,1;(..) | *19 | *11 | *08 |  |  |  |  |  | 0.027 |  |
| I | 10,11,13,15,16,27;17,1;(..) | *19 | *02 | *13 |  |  |  |  |  | 0.027 |  |
| I | 10,11,13,15,16,27;17,1;(..) | *19 | *11 | *03 |  |  |  |  |  | 0.027 |  |
| I | 10,11,13,15,16,27;17,1;(..) | *19 | *02 | *03 |  |  |  |  |  | 0.036 |  |
| I | 10,11,13,15,16,27;17,1;(..) | *19 | *02 | *12 |  |  |  |  |  | 0.009 |  |
| I | 10,11,13,15,16,27;17,1;(..) | *19 | *11 | *11 |  |  |  |  |  | 0.009 |  |
| I | 10,11,13,15,16,27;17,1;(..) | *19 | *13 | *03 |  |  |  |  |  | 0.009 |  |
| J | 5,10,11,13,14,26,27;3,1;23 | *11 | *08 | *02 |  |  |  | 0.010 |  |  |  |
| J | 5,10,11,13,14,26,27;3,1;23 | *21 | *08 | *02 |  |  |  |  |  | 0.109 |  |
| K | 5,10,11,13,14,26,27;3,1;(..) | *21 | *08 | *03 |  |  |  |  |  | 0.027 |  |
| K | 5,10,11,13,14,26,27;3,1;(..) | *21 | *08 | *13 |  |  |  |  |  | 0.018 |  |
|  | 21,27;17,1;(..) | *22 | *02 | *03 |  | 0.063 | 0.137 |  | 0.010 |  | 0.011 |
|  | 21,27;17,1;(..) | *22 | *14 | *08 |  |  | 0.010 |  |  |  |  |
|  | 21,27;17,1;(..) | *22 | *02 | *10 |  |  | 0.010 |  |  |  |  |
|  | 21,26,27,28;3;23 | *14 | *03 | *02 |  |  | 0.010 |  |  |  |  |
|  | 5,10,11,13,14,26,27;17,1;23 | *21 | *02 | *02 |  |  |  |  |  | 0.009 |  |
|  | 10,11,13,16,27;17,1;(..) | *23 | *02 | *08 |  |  |  |  |  | 0.009 |  |
|  | 5,10,11,13,14,26,27;17,1;(..) | *10 | *05 | *06 |  |  |  |  |  |  | 0.011 |
|  | 21,27;17,1,2;(..) | *22 | *07 | *03 |  |  |  |  |  |  | 0.023 |
|  | 5,10,11,13,14,26,27;17,1;(..) | *01 | *05 | *06 |  |  |  |  |  |  | 0.011 |

^1^ Allotype haplotype ID are as described in LEFRAC; LEFRAC, 2012.

Table S5: Statistical significance of codon-based test of selection between sequences of *IGHG1* gene segment

| *IGHG1* | **01* | **02* | **03* | **04* | **05* | **06* | **07* | **08* | **09* | **10* | **11* | **12* | **13* | **14* |
| --- | --- | --- | --- | --- | --- | --- | --- | --- | --- | --- | --- | --- | --- | --- |
| **01* |  | 0.160 | 0.135 | 0.262 | **0.043** | 0.080 | 0.262 | 0.262 | 0.080 | 0.080 | 0.066 | 0.080 | 0.262 | 0.074 |
| **02* | 1.000 |  | 0.252 | 1.000 | 0.080 | 0.160 | 1.000 | 1.000 | 0.160 | 0.160 | 0.126 | 0.160 | 1.000 | 0.135 |
| **03* | 1.000 | 1.000 |  | 0.329 | 0.074 | 0.135 | 0.329 | 0.184 | 0.135 | 0.135 | 0.101 | 0.135 | 0.328 | 0.160 |
| **04* | 1.000 | 0.160 | 1.000 |  | 0.126 | 0.262 | 1.000 | 1.000 | 0.262 | 0.262 | 0.184 | 0.262 | 1.000 | 0.181 |
| **05* | 1.000 | 1.000 | 1.000 | 1.000 |  | **0.043** | 0.126 | 0.126 | 0.160 | **0.043** | **0.036** | **0.043** | 0.126 | **0.042** |
| *06* | 1.000 | 1.000 | 1.000 | 1.000 | 1.000 |  | 0.262 | 0.262 | 0.080 | 0.080 | 0.066 | 0.080 | 0.262 | 0.074 |
| **07* | 1.000 | 0.160 | 1.000 | 0.080 | 1.000 | 1.000 |  | 1.000 | 0.262 | 0.262 | 0.184 | 0.262 | 1.000 | 0.181 |
| **08* | 1.000 | 0.160 | 1.000 | 0.080 | 1.000 | 1.000 | 0.080 |  | 0.262 | 0.262 | 0.184 | 0.262 | 1.000 | 0.097 |
| **09* | 1.000 | 1.000 | 1.000 | 1.000 | 1.000 | 1.000 | 1.000 | 1.000 |  | 0.080 | 0.066 | 0.080 | 0.262 | 0.074 |
| **10* | 1.000 | 1.000 | 1.000 | 1.000 | 1.000 | 1.000 | 1.000 | 1.000 | 1.000 |  | 0.066 | 0.080 | 0.262 | 0.074 |
| **11* | 1.000 | 1.000 | 1.000 | 1.000 | 1.000 | 1.000 | 1.000 | 1.000 | 1.000 | 1.000 |  | 0.066 | 0.184 | 0.058 |
| **12* | 1.000 | 1.000 | 1.000 | 1.000 | 1.000 | 1.000 | 1.000 | 1.000 | 1.000 | 1.000 | 1.000 |  | 0.262 | 0.074 |
| **13* | 1.000 | 0.160 | 1.000 | 0.080 | 1.000 | 1.000 | 0.080 | 0.080 | 1.000 | 1.000 | 1.000 | 1.000 |  | 0.180 |
| **14* | 1.000 | 1.000 | 1.000 | 1.000 | 1.000 | 1.000 | 1.000 | 1.000 | 1.000 | 1.000 | 1.000 | 1.000 | 1.000 |  |

Above the diagonal are the p-value of statistical test for purifying selection (dN < dS) and below the diagonal for positive selection (dN > dS).

P-values of less than 0.05 are considered significant and are in bold.

Table S6: Statistical significance of codon-based test of selection between sequences of *IGHG2* gene segment

| *IGHG2* | **01* | **02* | **03* | **04* | **05* | **06* | **07* | **08* | **09* | **10* | **11* | **12* | **13* | **14* | **15* |
| --- | --- | --- | --- | --- | --- | --- | --- | --- | --- | --- | --- | --- | --- | --- | --- |
| **01* |  | 0.052 | 0.160 | 0.371 | 0.080 | **0.036** | 0.080 | **0.024** | 0.181 | 0.080 | **0.036** | 0.080 | **0.043** | 0.066 | **0.036** |
| **02* | 1.000 |  | 0.095 | 0.175 | 0.052 | 1.000 | 0.180 | 0.180 | 0.098 | 0.052 | 0.246 | 0.180 | 0.095 | 0.485 | 0.246 |
| **03* | 1.000 | 1.000 |  | 1.000 | 0.160 | 0.066 | 0.160 | **0.043** | 0.372 | 0.160 | 0.066 | 0.160 | 0.080 | 0.124 | 0.066 |
| **04* | 1.000 | 1.000 | 0.080 |  | 0.371 | 0.132 | 0.371 | 0.095 | 1.000 | 0.371 | 0.132 | 0.371 | 0.180 | 0.247 | 0.132 |
| **05* | 1.000 | 1.000 | 1.000 | 1.000 |  | **0.036** | 0.080 | 0.080 | 0.181 | 0.080 | 0.124 | 0.080 | 0.160 | 0.066 | 0.124 |
| **06* | 1.000 | **0.043** | 1.000 | 1.000 | 1.000 |  | 0.124 | 0.124 | 0.073 | **0.036** | 0.180 | 0.124 | 0.066 | 0.371 | 0.180 |
| **07* | 1.000 | 1.000 | 1.000 | 1.000 | 1.000 | 1.000 |  | 0.080 | 0.181 | 0.080 | 0.124 | 0.080 | 0.160 | 0.259 | 0.124 |
| **08* | 1.000 | 1.000 | 1.000 | 1.000 | 1.000 | 1.000 | 1.000 |  | 0.052 | **0.024** | 1.000 | 0.080 | 0.160 | 0.259 | 1.000 |
| **09* | 1.000 | 1.000 | 1.000 | 0.406 | 1.000 | 1.000 | 1.000 | 1.000 |  | 0.181 | 0.073 | 0.181 | 0.095 | 0.132 | 0.073 |
| **10* | 1.000 | 1.000 | 1.000 | 1.000 | 1.000 | 1.000 | 1.000 | 1.000 | 1.000 |  | **0.036** | 0.080 | **0.043** | 0.066 | **0.036** |
| **11* | 1.000 | 1.000 | 1.000 | 1.000 | 1.000 | 1.000 | 1.000 | 0.160 | 1.000 | 1.000 |  | 0.124 | 0.259 | 0.160 | 1.000 |
| **12* | 1.000 | 1.000 | 1.000 | 1.000 | 1.000 | 1.000 | 1.000 | 1.000 | 1.000 | 1.000 | 1.000 |  | **0.043** | 0.259 | 0.124 |
| **13* | 1.000 | 1.000 | 1.000 | 1.000 | 1.000 | 1.000 | 1.000 | 1.000 | 1.000 | 1.000 | 1.000 | 1.000 |  | 0.124 | 0.259 |
| **14* | 1.000 | 1.000 | 1.000 | 1.000 | 1.000 | 1.000 | 1.000 | 1.000 | 1.000 | 1.000 | 1.000 | 1.000 | 1.000 |  | 0.371 |
| **15* | 1.000 | 1.000 | 1.000 | 1.000 | 1.000 | 1.000 | 1.000 | 0.160 | 1.000 | 1.000 | 0.080 | 1.000 | 1.000 | 1.000 |  |

Above the diagonal are the p-value of statistical test for purifying selection (dN < dS) and below the diagonal for positive selection (dN > dS).

P-values of less than 0.05 are considered significant and are in bold.

Table S7: Statistical significance of codon-based test of selection between sequences of *IGHG3* gene segment

| *IGHG3* | *01 | *02 | *03 | *04 | *06 | *07 | *08 | *09 | *10 | *11 | *13 | *14 | *15 | *16 | *17 | *18 | *19 | *20 | *21 | *22 | *23 | *24 | *25 | *26 | *27 | *28 | *29 |
| --- | --- | --- | --- | --- | --- | --- | --- | --- | --- | --- | --- | --- | --- | --- | --- | --- | --- | --- | --- | --- | --- | --- | --- | --- | --- | --- | --- |
| *01 |  | 0.160 | 0.327 | 0.160 | 0.125 | 0.260 | 0.260 | 0.260 | 0.160 | 1.000 | 0.374 | 0.491 | 1.000 | 1.000 | 1.000 | 1.000 | 1.000 | 1.000 | 0.160 | 1.000 | 1.000 | 0.490 | 0.326 | 1.000 | 0.250 | 0.260 | 0.250 |
| *02 | 1.000 |  | 0.180 | 0.080 | 0.066 | 0.125 | 0.125 | 0.125 | 0.080 | 0.260 | 0.182 | 0.250 | 0.326 | 0.326 | 1.000 | 1.000 | 1.000 | 0.326 | 0.080 | 0.325 | 1.000 | 0.250 | 0.179 | 0.374 | 0.134 | 0.125 | 0.134 |
| *03 | 1.000 | 1.000 |  | 0.180 | 0.133 | 0.232 | 0.232 | 0.232 | 0.180 | 0.406 | 0.180 | 0.355 | 0.421 | 0.421 | 1.000 | 1.000 | 1.000 | 0.421 | 0.180 | 0.420 | 1.000 | 0.355 | 0.263 | 0.487 | 0.215 | 0.232 | 0.215 |
| *04 | 1.000 | 1.000 | 1.000 |  | 0.066 | 0.125 | 0.125 | 0.125 | 0.080 | 0.260 | 0.182 | 0.250 | 0.326 | 0.326 | 1.000 | 1.000 | 1.000 | 0.326 | 0.080 | 0.325 | 1.000 | 0.250 | 0.179 | 0.374 | 0.134 | 0.125 | 0.134 |
| *06 | 1.000 | 1.000 | 1.000 | 1.000 |  | 0.160 | 0.096 | 0.096 | 0.260 | 0.182 | 0.066 | 0.179 | 0.134 | 0.231 | 0.485 | 0.483 | 0.403 | 0.232 | 0.066 | 0.231 | 0.324 | 0.096 | 0.132 | 0.249 | 0.100 | 0.096 | 0.326 |
| *07 | 1.000 | 1.000 | 1.000 | 1.000 | 1.000 |  | 0.182 | 0.182 | 0.125 | 0.374 | 0.125 | 0.326 | 0.250 | 0.406 | 1.000 | 1.000 | 1.000 | 0.406 | 0.125 | 0.405 | 1.000 | 0.182 | 0.231 | 0.489 | 0.179 | 0.182 | 1.000 |
| *08 | 1.000 | 1.000 | 1.000 | 1.000 | 1.000 | 1.000 |  | 0.182 | 0.125 | 0.374 | 0.249 | 1.000 | 1.000 | 1.000 | 1.000 | 1.000 | 1.000 | 1.000 | 0.125 | 1.000 | 1.000 | 1.000 | 0.491 | 0.489 | 0.375 | 0.182 | 0.375 |
| *09 | 1.000 | 1.000 | 1.000 | 1.000 | 1.000 | 1.000 | 1.000 |  | 0.125 | 0.373 | 0.249 | 0.325 | 0.405 | 0.405 | 1.000 | 1.000 | 1.000 | 0.406 | 0.125 | 0.405 | 1.000 | 0.325 | 0.231 | 0.489 | 0.179 | 0.182 | 0.179 |
| *10 | 1.000 | 1.000 | 1.000 | 1.000 | 1.000 | 1.000 | 1.000 | 1.000 |  | 0.260 | 0.182 | 0.250 | 0.326 | 0.326 | 1.000 | 1.000 | 1.000 | 0.326 | 0.080 | 0.325 | 1.000 | 0.250 | 0.179 | 0.374 | 0.134 | 0.125 | 0.134 |
| *11 | 0.160 | 1.000 | 1.000 | 1.000 | 1.000 | 1.000 | 1.000 | 1.000 | 1.000 |  | 0.489 | 1.000 | 1.000 | 1.000 | 1.000 | 1.000 | 1.000 | 1.000 | 0.260 | 1.000 | 1.000 | 1.000 | 0.405 | 1.000 | 0.325 | 0.160 | 0.325 |
| *13 | 1.000 | 1.000 | 1.000 | 1.000 | 1.000 | 1.000 | 1.000 | 1.000 | 1.000 | 1.000 |  | 0.406 | 0.326 | 0.487 | 1.000 | 1.000 | 1.000 | 0.487 | 0.182 | 0.486 | 1.000 | 0.250 | 0.290 | 1.000 | 0.231 | 0.249 | 0.231 |
| *14 | 1.000 | 1.000 | 1.000 | 1.000 | 1.000 | 1.000 | 0.080 | 1.000 | 1.000 | 0.402 | 1.000 |  | 1.000 | 1.000 | 1.000 | 1.000 | 1.000 | 1.000 | 0.250 | 1.000 | 1.000 | 1.000 | 0.261 | 1.000 | 0.160 | 0.325 | 0.160 |
| *15 | 0.402 | 1.000 | 1.000 | 1.000 | 1.000 | 1.000 | **0.043** | 1.000 | 1.000 | 0.308 | 1.000 | 0.160 |  | 1.000 | 1.000 | 1.000 | 1.000 | 1.000 | 0.326 | 1.000 | 1.000 | 1.000 | 0.376 | 1.000 | 0.261 | 0.405 | 0.261 |
| *16 | 0.402 | 1.000 | 1.000 | 1.000 | 1.000 | 1.000 | **0.043** | 1.000 | 1.000 | 0.308 | 1.000 | 0.160 | 0.080 |  | 1.000 | 1.000 | 1.000 | 1.000 | 0.326 | 1.000 | 1.000 | 1.000 | 0.376 | 1.000 | 0.261 | 0.405 | 0.261 |
| *17 | **0.005** | 0.169 | 0.295 | 0.169 | 1.000 | 0.231 | 0.121 | 0.122 | 0.169 | **0.003** | 0.169 | 0.120 | 0.168 | 0.085 |  | 1.000 | 1.000 | 1.000 | 1.000 | 1.000 | 1.000 | 1.000 | 1.000 | 1.000 | 1.000 | 1.000 | 1.000 |
| *18 | **0.005** | 0.170 | 0.296 | 0.170 | 1.000 | 0.232 | 0.122 | 0.122 | 0.170 | **0.003** | 0.170 | 0.121 | 0.169 | 0.086 | **0.024** |  | 1.000 | 1.000 | 1.000 | 1.000 | 1.000 | 1.000 | 1.000 | 1.000 | 1.000 | 1.000 | 1.000 |
| *19 | **0.008** | 0.232 | 0.362 | 0.232 | 1.000 | 0.310 | 0.170 | 0.170 | 0.232 | **0.005** | 0.232 | 0.169 | 0.231 | 0.121 | **0.043** | 0.160 |  | 1.000 | 1.000 | 1.000 | 1.000 | 1.000 | 1.000 | 1.000 | 1.000 | 1.000 | 1.000 |
| *20 | 0.401 | 1.000 | 1.000 | 1.000 | 1.000 | 1.000 | **0.043** | 1.000 | 1.000 | 0.308 | 1.000 | 0.160 | 0.080 | 0.080 | 0.085 | 0.086 | 0.121 |  | 0.325 | 1.000 | 1.000 | 1.000 | 0.376 | 1.000 | 0.261 | 0.405 | 0.261 |
| *21 | 1.000 | 1.000 | 1.000 | 1.000 | 1.000 | 1.000 | 1.000 | 1.000 | 1.000 | 1.000 | 1.000 | 1.000 | 1.000 | 1.000 | 0.169 | 0.170 | 0.232 | 0.080 |  | 0.326 | 1.000 | 0.250 | 0.179 | 0.374 | 0.134 | 0.125 | 0.134 |
| *22 | 0.402 | 1.000 | 1.000 | 1.000 | 1.000 | 1.000 | **0.043** | 1.000 | 1.000 | 0.309 | 1.000 | 0.160 | 0.080 | 0.080 | 0.169 | 0.169 | 0.232 | 1.000 | 1.000 |  | 1.000 | 1.000 | 0.375 | 1.000 | 0.261 | 0.404 | 0.261 |
| *23 | **0.014** | 0.309 | 0.434 | 0.309 | 1.000 | 0.403 | 0.231 | 0.231 | 0.309 | **0.008** | 0.309 | 0.230 | 0.308 | 0.168 | **0.024** | 0.080 | 0.160 | 0.168 | 0.309 | 0.309 |  | 1.000 | 1.000 | 1.000 | 0.486 | 1.000 | 0.486 |
| *24 | 1.000 | 1.000 | 1.000 | 1.000 | 1.000 | 1.000 | 0.080 | 1.000 | 1.000 | 0.403 | 1.000 | 0.080 | 0.160 | **0.043** | 0.230 | 0.231 | 0.309 | **0.043** | 1.000 | **0.043** | 0.402 |  | 0.491 | 1.000 | 0.375 | 0.325 | 0.375 |
| *25 | 1.000 | 1.000 | 1.000 | 1.000 | 1.000 | 1.000 | 1.000 | 1.000 | 1.000 | 1.000 | 1.000 | 1.000 | 1.000 | 1.000 | 0.296 | 0.298 | 0.364 | 1.000 | 1.000 | 1.000 | 0.436 | 1.000 |  | 0.486 | 0.125 | 0.231 | 0.125 |
| *26 | 0.080 | 1.000 | 1.000 | 1.000 | 1.000 | 1.000 | 1.000 | 1.000 | 1.000 | 0.160 | 0.403 | 0.308 | 0.230 | 0.230 | **0.002** | **0.002** | **0.003** | 0.230 | 1.000 | 0.231 | **0.005** | 0.309 | 1.000 |  | 0.405 | 0.260 | 0.405 |
| *27 | 1.000 | 1.000 | 1.000 | 1.000 | 1.000 | 1.000 | 1.000 | 1.000 | 1.000 | 1.000 | 1.000 | 1.000 | 1.000 | 1.000 | 0.362 | 0.364 | 0.437 | 1.000 | 1.000 | 1.000 | 1.000 | 1.000 | 1.000 | 1.000 |  | 0.179 | 0.080 |
| *28 | 1.000 | 1.000 | 1.000 | 1.000 | 1.000 | 1.000 | 1.000 | 1.000 | 1.000 | 1.000 | 1.000 | 1.000 | 1.000 | 1.000 | 0.122 | 0.123 | 0.170 | 1.000 | 1.000 | 1.000 | 0.232 | 1.000 | 1.000 | 1.000 | 1.000 |  | 0.179 |
| *29 | 1.000 | 1.000 | 1.000 | 1.000 | 1.000 | 0.402 | 1.000 | 1.000 | 1.000 | 1.000 | 1.000 | 1.000 | 1.000 | 1.000 | 0.362 | 0.364 | 0.437 | 1.000 | 1.000 | 1.000 | 1.000 | 1.000 | 1.000 | 1.000 | 1.000 | 1.000 |  |

Above the diagonal are the p-value of statistical test for purifying selection (dN < dS) and below the diagonal for positive selection (dN > dS).

P-values of less than 0.05 are considered significant and are in bold.
